# Development and Validation of the Young Parenting Inventory (YPI-R3) for Measuring Past Deviant and Normal Variations in Parenting

**DOI:** 10.3390/children9050706

**Published:** 2022-05-11

**Authors:** John P. Louis, Karen M. Louis, George Lockwood

**Affiliations:** 1 Louis Counselling & Training Services Pte. Ltd., Singapore 339159, Singapore; kmclouis@gmail.com; 2 Schema Therapy Institute Midwest, Kalamazoo, MI 49009, USA; glockwood@chartermi.net

**Keywords:** parenting, deviant, cross-culturally, schemas, factor analysis

## Abstract

Most measures of past parenting patterns have a restricted range of about two to three negative parenting constructs. The Young Parenting Inventory (YPI-R2) provides a more nuanced framework that measures a fuller spectrum of these negative parenting patterns and, therefore, holds the potential of being a more useful guide to parents and caretakers. The YPI-R2 is made up of six validated subscales. An additional four were identified but were not sufficiently robust to be included. The purpose of this study is to determine if these four scales can be strengthened through the development of additional items and be empirically validated. Using non-clinical, English-speaking community samples from Singapore (*n* = 592, 628) and Malaysia (*n* = 222, 229), these revised scales were tested using multiple exploratory factor analyses with fathers and mothers rated separately. After further scale refinement, the final model, which consisted of 10 subscales and 41 items, was then subjected to confirmatory factor analysis using 4 other non-clinical international samples with separate ratings for fathers and mothers—USA (*n* = 259, 281), South Africa (*n* = 318, 372), Nigeria (*n* = 328, 344) and India (*n* = 277, 289). The results show that the YPI-R3 with 10 subscales is a robust and cross-culturally acceptable model. Correlations and hierarchical multiple regression analyses showed that the YPI-R3 has good convergent validity and predictive capabilities with measures of psychopathology, personality traits, emotional distress, negative schemas and other distal measures of functioning in everyday life—gratitude, humor and satisfaction with life.

## 1. Public Significance Statement

Other models and measures of past negative parenting patterns are mostly made up of two to three constructs. This study developed a new instrument that identifies and measures 10 unhealthy parenting patterns. These patterns were linked positively with ill-being, and negative schemas (distinct thinking patterns and experiences), but negatively with well-being.

## 2. Introduction

Research in parent–child interactions and their outcomes in children began just over seven decades ago, when Baldwin et al. [1] conducted a study in 1945 that identified two parenting constructs—Autocratic and Democratic. In the 1960s, influenced by Baldwin [1], Baumrind [2] used qualitative analysis to uncover three parenting styles. Later, Maccoby and Martin [3] added a fourth parenting style; all of these four parenting styles were based on variations of warmth and control and were labeled as Authoritative (high warmth-high control), Authoritarian (low warmth-high control), Permissive (high warmth-low control), and Neglectful (low warmth-low control). Parenting styles were defined by Darling and Steinberg [4] as “a combination of various elements that create an emotional climate in which parents communicate their attitudes and practices about childrearing to their child”. Numerous studies for over 60 years revealed that these four parenting constructs are associated with children’s externalizing problems and academic achievements [4]. However, Baumrind’s [2,3] broad parenting constructs were based on normal variations of parenting used to control and socialize children that did not include deviant parenting practices, such as abuse and neglect [5,6]. These which have been linked to a range of dysfunctional outcomes, including personality disorders [7]. Therefore, there is a need to explore parenting dimensions beyond Baumrind’s typology in order to include a fuller spectrum of maladaptive parenting constructs associated with psychopathology. 

### 2.1. Theoretical Framework

Since Baumrind’s model [2,3] was based on normal variations in parenting, we describe these normal variations as being predictable and expectable for a substantial number of families in a society. Baumrind’s model was derived qualitatively. A readily accessible standardized qualitative measure was not available until Buri [8] developed the Parenting Authority Questionnaire (PAQ). The PAQ is a Likert scale that measures the three parenting styles of Baumrind’s typology. The items in the PAQ are appraised by the children of the parents in question [8]. An example of an item that represents the Authoritarian parenting style from Baumrind’s model in the PAQ is: “Even if his/her children didn’t agree with him/her, my father/mother felt that it was for our own good if we were forced to conform to what he/she thought was right” [8]. An item example for the Permissive parenting style from PAQ is: “While I was growing up my father/mother felt that in a well-run home children should have their way in the family as often as the parents do” [8]. Outside the framework of these types of normative parenting styles, there are parenting patterns that are more harmful and less normative that reflect abuse or gross neglect. Such dysfunctional patterns have been known to be linked to a range of psychopathology, including personality disorders [9,10,11]. Examples of items that represent this kind of parenting patterns are: “Abused me physically: did things like calling me names, screaming at me, swearing at me, or threatening me”; “Seemed to get pleasure out of hurting me”; “Kept our family isolated from others”; “Took money or a possession from me against my wishes to use for him/herself”; “Put me down and made me feel ashamed of myself if I didn’t do well”. While cultures with healthy parenting paradigms would not condone such clearly abusive or deviant parental behaviors, a few parental behaviors are, on the other hand, culturally sensitive in that they may be regarded as normal or deviant in one culture but not in another. A prime example is administering physical punishment or “spanking”, which has been used by the majority of Americans as well as many Asians and African parents at some point in their parenting history [12]. In many Asian and African countries, such physical discipline is viewed as being normative, while in other countries, such as those in Scandinavia, it is frowned upon and even outlawed [13]. Thus, while clear cut and severe abusive parenting practices are likely to gain universal acceptance, the point at which parenting patterns cross from normative to deviant cannot be accepted universally. Notwithstanding the cultural variations in some aspects of parenting, from a scientific vantage point, all forms of parenting styles and or practices that result in unfavorable outcomes in children are deemed maladaptive regardless of whether these are normative practices or deviant. 

The Young Parent Inventory (YPI, [7]) is arguably the most comprehensive scale for measuring maladaptive parenting patterns, but its psychometric soundness has never been proven. The YPI was developed from the vantage point of schema therapy and was hypothesized to be associated with the development of negative schemas and pathology [7,14,15]. Rather than having just two or three broad parenting patterns as postulated by Baumrind [2,3], the YPI hypothesized 17 maladaptive parenting patterns representing a fuller range, including normal and deviant practices. Since the YPI had not been validated, an initial item pool, which consisted of 204 past negative parenting items (72 items from the original YPI and 132 new items), was developed by Louis et al. [15]. This item pool led to the development of a validated version of the YPI, known as YPI-R2, which consisted of 6 subscales and 36 items [15]. However, four subscales that represented more deviant parenting patterns were rejected during this developmental process. Therefore, it was believed that further item development might lead to the inclusion of the rejected subscales, which would result in a more comprehensive scale measuring a greater range of maladaptive and deviant parenting constructs. This study set out to test this hypothesis with the potential of it leading to the development of a validated and a more comprehensive scale that would be known as the YPI-R3. 

### 2.2. Limitations of the Current Scales Measuring Past Maladaptive Parenting Patterns

The small number of parenting constructs that constitute Baumrind’s parenting typology was highlighted as a limitation [16,17,18,19]. Grolnick [20] and Greenspan [21] disagreed with Baumrind’s view that high control should be part of her Authoritative parenting construct based on Attribution theory [22]—that this would prevent children from clearly differentiating their behaviors from their internal desires. Other parenting measures assessing past maladaptive parenting behaviors, also have similar limitations in that only a few constructs were developed. Examples of some of the most widely used ones include the s-EMBU (Swedish acronym for “My memories of upbringing”), which has two maladaptive subscales: Rejection, and Overprotection [23]. The Childhood Trauma Questionnaire (CTQ) has five maladaptive subscales [24], but these are based on only two broader constructs, Abuse and Neglect. The Parental Acceptance–Rejection Questionnaire (PARQ, Adult version) has three maladaptive ones, representing Rejection called Hostility, Indifferent and Undifferentiated [25]. The Alabama Parenting Questionnaire [26] has three involving control called Poor Monitoring, Inconsistent Discipline, and Corporal Punishment. The Parker Bonding Instrument has one adaptive and one maladaptive construct, called Care and Overprotective, respectively [27]. The Measure of Parental Style has three maladaptive constructs labeled Indifference, Abuse and Overcontrol [28]. Given the complexity of childhood development and variations in needs among children in different developmental phases, it seems more likely that maladaptive parent–child interactions are a complex process that cannot be reduced to only a few constructs. For example, the maladaptive construct of “Rejection” is popularly used in many of the scales mentioned above. A child may perceive Rejection in a variety of ways, such as feeling disliked, not given attention, being harshly punished for trivial offences, being shamed in front of others constantly, being derogatorily labeled, being put down, receiving the brunt of parents’ anger, being a burden, not feeling special, or being abandoned at a young age. It follows that parents and caregivers may gain deeper insights and understanding from a model that encapsulates a wide range of parent–child patterns that can, for example, delineate the many facets that make up a broad construct such as Rejection. Furthermore, such nuanced and more dysfunctional parenting patterns have been found to contribute to the development of forms of pathology, such as personality disorders, as highlighted by Young et al. [7], Agrawal et al. [9], Bandelow et al. [10] and Schuppert et al. [11]. These include negative patterns, such as invalidation of children’s emotions, abuse, neglect, overprotection (e.g., mothers who are overly dependent on their children emotionally), environment instability (frequent changes in housing and schooling) and high levels of distress and frustration on the part of the parent. These types of patterns need to be included if one intends to capture the full spectrum of maladaptive parenting. While the YPI-R2 (with six subscales) demonstrated construct, convergent, divergent and incremental validity [19], four subscales were not robust enough to be included. It was hypothesized that these scales could be strengthened by adding items that better captured the underlying constructs associated with each, leading to a yet more comprehensive measure of dysfunctional parenting patterns since other established instruments are limited in the range of their constructs, measuring only two to three past negative parenting patterns. This study was designed to test this hypothesis. 

### 2.3. The Present Research

This research is conducted in two stages: Study 1 and Study 2. The aim of Study 1 is to develop an improved version of the YPI-R2, known as YPI-R3, by adding more robust items to the four weaker subscales (Intrusiveness and Exploitation, Undependability and Irresponsibility, “Social Exclusion”, and “Fear of Harm and Illness”; [15]. These weaker subscales likely caused the fit indices to not reach the recommended cut off points during the development of the YPI-R2, evidenced by the fact that when they were removed the fit indices improved [15]. As a result, these were removed and only six subscales were accepted into the final version of the YPI-R2 [15]. It was hypothesized that the reason for the rejection of these subscales was due to items not being sufficiently robust. These constructs and items were examined by the expert team and the decisions reached were as follows: Three new items for the subscales of Intrusiveness and Exploitation, Social Exclusion, and Undependability and Irresponsibility were developed and added (see Supplemental Information, Table S2). However, no new items were added for the Fear of Harm and Illness subscale as it was deemed to be well represented clinically with four items (see Supplemental Information, end of Table S1). Moreover, by examination of face validity, several items that represented Fear of Harm and Illness were similar to items that represented the Overprotection subscale (which was already well represented), such as “Worried excessively that I would get hurt” and “Worried excessively that I would get sick”, and the expert team was careful to not introduce any redundant subscales (see Supplementary Materials, Table S1 under both subscales). Additionally, it is noteworthy that, since the robustness of the subscale of Over-Control (previously labeled as Controlling) was also questionable during the development of the YPI-R2 (see page 12 of Louis et al. [15]), the expert team also decided to strengthen this subscale to ensure that it emerged strongly and therefore three more items were added (See Supplementary Materials, Table S2). One item belonging to the subscale of Competitiveness and Status Seeking, namely “Believed it was more important for me to gain wealth and status than be true to myself”, was also added as this new item was considered to be clinically relevant in that many clients were driven by such messages from their parents. Moreover, there was a need to provide a more balanced representation of the subscale of “Overprotection and Overindulgent”. In the current version of the YPI-R2, this subscale was imbalanced in that, out of its eight items, only two items reflected the Overindulgent construct, while the other six represented the Overprotection construct. This imbalance was likely due to the items representing “Overindulgence” not being sufficiently robust (See Supplementary Materials, Table S1 under the subscale Overprotection and Overindulgence). Thus, in order to maximize the chance of at least three items representing the construct of Overindulgence to appear, 10 new items that reflect this construct were added. Another reason for adding more items to help the construct of Overindulgence to appear was that past studies conducted to bring out similar constructs, such as the negative schema of Insufficient Self-Control, which was hypothesized by Young to originate from indulgent parenting patterns, have had challenges in emerging robustly [29,30], including the study where the YPI-R2 was developed [15] in which only two items representing Overindulgence emerged. The addition of these items brought the total number of new items to 23 (See Supplementary Materials, Table S2). In summary, the addition of these 23 items were based partly on empirical justification—the identification of the weaker subscales of the YPI-R2 [15] and those that historically did not emerge robustly—as well as involving judgement calls by the expert team. These 23 new items were then added to the 51 items that represent the other six robust subscales of the YPI-R2, bringing the total to 74 items (see Supplementary Materials, bottom of Table S1). These items were then subjected to multiple exploratory factor analyses (EFAs) in Study 1 using two Asian samples drawn from non-clinical, English-speaking community populations in Singapore and Malaysia. It was hypothesized that the weaker subscales rejected during the development of the YPI-R2 [15] would become sufficiently robust to be included in the newly improved scale, known as the YPI-R3. It was also hypothesized that all current six subscales of the YPI-R2 would be replicated and retained in the YPI-R3. A comprehensive scale with as many as ten constructs measuring past parenting patterns would enable clinicians to more fully assess both normal and deviant parenting patterns since other established instruments are greatly limited in the range of their constructs and measure only two to three patterns [7,9,10,11]. 

The first aim of Study 2 is to subject the YPI-R3 scale to confirmatory factor analysis (CFA) using four other international non-clinical English-speaking community samples—USA, South Africa, Nigeria and India. It was hypothesized that the YPI-R3 model would prove to be a cross-culturally acceptable one. The second and final aim of Study 2 is to test for convergent validity by subjecting the YPI-R3 scale to psychometric testing using other established measures of psychopathology, personality traits, emotional distress, negative schemas and other distal measures of functioning in everyday life. 

## 3. Method

### Samples

Six multicultural community samples (non-clinical) fluent in the English language were used in this study—Singapore, Malaysia, the USA, South Africa, Nigeria and India. The (*n =* sample size), mean age and standard deviation (SD) of the samples were as follows: the USA: (*n* = 396), 43.69 years, SD = 9.12; South Africa: (*n* = 390), 42.11 years, SD = 6.79; Nigeria: (*n* = 364), 45.7 years, SD = 7.19; India: (*n* = 306), 42.39 years, SD = 7.67; Singapore: (*n* = 628), 46.22 years, SD = 22.32; and Malaysia: (*n* = 229), 41.40, SD = 17.40. Since participants had to provide separate ratings for their fathers and mothers, the above six samples resulted in 12 separate samples. Table 1 shows the demographic characteristics of these samples. 

Data from the Singapore and Malaysia samples were obtained from a research study conducted in 2016 using self-report questionnaires from participants attending a community, in-person parenting workshop. For the other four samples, data were obtained online during the COVID-19 outbreak in 2020 as in-person meetings were disallowed on a global scale. Participation was not excluded on the basis of religion, color or race and was completely voluntary. Endorsement of this survey was given by the NGO in each city. These volunteers were given time to consider participating and providing informed consent as notice was given to them eight weeks ahead of time by emails. The notice highlighted the following information: that the survey was voluntary; that participants had to be at least 18 years of age; that they were currently in a marriage relationship (this information was used for a separate study); and that they were sufficiently fluent in English to be able to understand items from a survey. The participants were also assured of the confidentiality and anonymity of the data. Participants were also informed that the purpose of the research was for scientific publications in the field of schemas, marriage and parenting. 

The same criteria were used for participants from Singapore and Malaysia, except that they did not have to be married. As a result, younger volunteers were able to participate, so the SD was larger for these two samples (see Table 1). These participants also invited their friends, who, in turn, invited others. This created a ripple effect and many attended this parenting workshop. Different incentives were given for the participants; those from Singapore and Malaysia met in a large and quiet meeting room and were given a book on parenting, and allowed to attend a no-charge parenting workshop after the survey. The online participants from the other countries were allowed to attend a two-hour parenting webinar without charge after completing the survey. However, participants who were not able to complete the survey or single parents who were not currently in a marriage relationship were not denied of these incentives. The ethical standards of this study were guided by principles set forth by the American Psychological Association and the British Psychological Society.

Participants who completed the survey online logged in three weeks before they received the incentive of the online parenting webinar. However, participants were given the choice to also complete the online survey from the comfort of their home. Logging in collectively gave them an advantage to raise any questions that they may have to their group leaders. These leaders were also present online with the participants and were briefed beforehand by the principal investigator of this research study. For the online survey, unanswered questions were flagged that reminded participants to return and respond to them. If the questions caused too much distress, then participants were allowed to abort the survey. 

Both the online and in-person surveys took 45 min to 1 h to complete. The responses from the survey were sent to the principal investigator and his assistant. All fields that were able to identify a participant, including IP addresses for the online participants, were not disclosed when the data were finally analyzed. 

## 4. EFA and Item Selection Process for the YPI-R3

### 4.1. Measures 

The following measures were administered for the Singapore and Malaysia samples only for Study 1. 

### 4.2. YPI-R2

The YPI-R2 [15] has 6 subscales and 36 items that measure perceived past parenting experiences. It uses a Likert scale with a range from 1 (*Completely untrue*) to 6 (*Describes him/her perfectly*). Examples of items include “Put a lot of emphasis on my getting good grades and getting ahead in life” (Competitiveness and Status Seeking subscale), and “Had a hard time being playful” (Emotional Inhibition and Deprivation subscale). The initial YPI [7] was developed based on the hypothesis that each negative schema was associated with a specific maladaptive parent–child interaction. The YPI-R2 scale was an improvement on the unvalidated YPI [15]. Separate ratings were obtained for father and mother. If a participant had another caregiver who assumed such a parental role—grandmother, grandfather, step-mother, step-father, older sibling—then these figures would be rated instead of their birth parent. This scale demonstrated construct validity with proven scales also measuring past parenting patterns—the s-EMBU (Swedish acronym for “My Memories of Upbringing”), Childhood Trauma Questionnaire (CTQ) and Parental Acceptance–Rejection Questionnaire Adult Version (PARQ)—where the average statistically significant correlation values were 0.30, 0.29 and 0.42, respectively [15]. The average correlation with personality traits, emotional distress, gratitude and Ryff’s well-being scale were 0.20, 0.19, 0.17 and 0.18, respectively [15]. The reliability values were mostly above 0.70 except for the Emotional Inhibition and Deprivation schema, and Overprotection and Overindulgence subscale, which were within the 0.60 to 0.70 range. The scale also showed incremental validity by accounting for additional significant variance in 12 out of 17 dependent variables over and above gender and the s-EMBU, CTQ and PARQ. The CFA results showed adequate fit values of 0.90 for the comparative and Tucker–Lewis Indices. Multi-group CFA was achieved at two out of the seven invariance levels [15]. While the YPI-R2 showed adequate psychometric testing for its six sub-subscales, four others were not deemed sufficiently robust to be included. Study 1 was focused on the development of new items in an effort to strengthen these weaker subscales. 

## 5. Procedures and Statistical Analyses for Study 1

IBM SPSS Statistics 23 (IBM Corp, Armonk, NY, USA, [31]) and M*Plus* 8 software [32] were used to conduct all analyses. For Study 1, samples from Singapore and Malaysia were used and participants with more than 10% missing data were deleted from the study. The analysis of missing data was conducted using the Little’s Missing Completely at Random (MCAR) test [33], which would ascertain the percentage of missing data as well as determine if missing patterns were at random. Since all sample sizes were > 200 (see Table 1), values of skewness and Kurtosis will not likely affect both CFA and EFA processes as both CFA and EFA will be robust against potential violations [34,35]. Moreover, Schafer [36] stated that a 5% level of missing data does not have a significant impact on the results.

Before reporting the results of the EFA, the data were analyzed to see if statistically significant differences of the mean age between men and women existed using a t-test for all six samples used for Study 1 and Study 2. The data were also analyzed to see if statistically significant differences existed in sample sizes and demographics of race and gender. Different sets of tests need to apply for different types of comparisons as there is no single test that can be applied to compare average values, proportions and proportions between groups [37,38]. A t-test is applied to compare averages between two groups (age); a z-test is the most suitable when comparison is made between proportions (men and women sample sizes); a chi-squared is used when comparison is made between several groups (different races, men and women) [37]. 

Study 1 began with the item development and selection stage in order to strengthen primarily the four weaker subscales of the YPI-R2, which were rejected during its development. These four weaker subscales were identified as Intrusiveness and Exploitation, Undependability and Irresponsibility, Social Exclusion, and Fear of Harm and Illness. The robust items that represented the six subscales of the YPI-R2 along with the less robust items that belonged to the four rejected subscales were selected, which totaled 51 items (see Supplementary Materials, Table S1; this table was published initially by Louis et al. [15], for the development of the YPI-R2). New items, totaling 23, were developed by an expert team to capture these weaker constructs of the YPI-R2 more accurately, as well as to strengthen others (see Supplementary Materials, Table S2; [15]). As a result, these 23 items were added to the chosen 51 items, which totaled 74 items representing 10 subscales. These 74 items were then administered to the Singapore and Malaysia samples with separate ratings for fathers and mothers for EFAs (See Supplementary Materials at the bottom of Table S2). 

Principal axis factoring (PAF) with promax rotation was used to perform the EFA. EFA was used instead of CFA at this stage as there were no firm theoretical factor structure as a result of having added new items to the YPI-R2 scale. EFA was conducted on both the Malaysia and Singapore samples to ensure that the factor structure that would emerge would not be based on just one, but multiple samples [14,15]. The number of factors to be extracted during an EFA was determined using a reliable method called Parallel Analysis (PA) [39]. During the EFAs, items that did not load > 0.40 were excluded [40], and items that had loadings > 0.40 on more than one factor were also removed. A further set of criteria were used to select the most robust items from the two EFAs, each with separate ratings for fathers and mothers (therefore four samples). They were: (1) items that loaded strongly in both the fathers and mothers samples from Singapore and Malaysia were retained [14,23]; (2) if a lower loading item was very similar in content to a higher loading item, then the higher loading item would be given priority and retained, unless the lower loading item was judged to have a greater clinical significance; (3) if an item appeared under one factor, for example, in the Singapore fathers sample, but not in the same factor in the Malaysia sample, then this item, if deemed to represent the construct precisely, would be chosen. Therefore, for some of the items, statistical rigor was balanced with its clinical significance and a certain degree of discretion was used by the expert team [14,40]. To ensure good fit indices at the later CFA stage, other items, such as those with loadings > 0.50, those with high modification indices, and with low average variance extracted (AVE) values, became targets for removal as recommended by Hair et al. [37] and Awang [38]. Furthermore, it was aimed to have a minimum of three, but up to six robust items per factor in the final YPI-R3 version as too many items in each factor would complicate the CFA process [40] and lengthen the scale unnecessarily. 

## 6. Results for Study 1

There were no missing data for the online survey from the USA, South Africa, Nigeria and India samples. For the sample from Malaysia (*n* = 222, 229), the percentage of missing data was 0.07%. The results of Little’s MCAR were as follows: test *X^2^* = 0.000, *df* = 16,494, *p* = 1.000. This shows that missing data were MCAR. However, the percentage of missing data for the Singapore sample (*n* = 592, 628) was 0.06%, but the results were strangely not MCAR (*X^2^* = 50,394.75, *df* = 48,588, *p* < 0.001), although such a phenomenon can happen in larger samples. Regardless, the effects of low missing rates (<5%) would be inconsequential [36]. 

### 6.1. Statistical Differences between Men and Women in Samples

The average age for men was significantly different than that for women for the USA, South Africa and Nigeria samples, but differences were insignificant for the India, Singapore and Malaysia samples. The proportion of women was significantly larger in the USA, South Africa, Nigeria, Singapore and Malaysia samples, but not for the sample from India. This may likely be due to more women being drawn to attend a workshop on parenting than men [41]. This was also the case for the fathers’ and mothers’ samples since separate ratings were obtained for both groups. The demographics for race did not differ significantly between men and women in all samples (see Supplementary Materials, Text S1). 

### 6.2. EFA and Item Selection

The number of factors extracted using PA from the Singapore fathers’ and mothers’ samples was 12 (accounted for 47% of the total variance) and 16 factors (accounted for 51% of the total variance), respectively. In the fathers’ factor solution, there was one factor that had only one item, so this factor was rejected. Another factor contained four items, two cross-loaded with a more robust factor labeled “Over-Control”, and given their vast overlap in face validity, this factor was also rejected. A new factor emerged, labeled as “Neglect and Insufficient Guidance”, which consisted of three robust items. For the mothers’ sample, seven factors had two items or less, and these were rejected, leaving behind nine factors. For the Malaysia sample, PA recommended 10 factors for the fathers’ (accounted for 47% of the variance) sample and nine factors (accounted for 46% of the variance) for the mothers’ sample. For the fathers’ sample, three factors had two items or less and were rejected. For the mothers’ sample, one factor had only one item and was rejected (see Supplementary Materials, Table S3, for factor structures of the most robust items). Since four-factor solutions emerged in total from the Singapore and Malaysia samples, the aforesaid criteria were used to select the most suitable items for the YPI-R3 scale. The final selection of the items from all four-factor solutions resulted in a model that consisted of 11 factors and 57 items (see Supplementary Materials, Table S3, items marked “✓”). These factors were labeled as Degradation and Rejection, Competitiveness and Status Seeking, Emotional Inhibition and Deprivation, Over-Control, Undependability and Irresponsibility, Overprotection Overindulgence, Insufficient Guidance and Neglect, Fear of Harm and Illness, Intrusiveness and Exploitation, Social Exclusion, and Punitiveness and Abuse. This 11-factor solution contained all the 6 subscales of the YPI-R2. However, 5 more new subscales emerged as a result of including 23 additional items. This model, known as the YPI-R3, was then subjected to the next stage of scale refinement.

### 6.3. Further Scale Refinement 

The initial model of 11 factors and 57 items in both fathers’ and mothers; samples from Singapore and Malaysia were subjected to CFA as a reference to assess the model fit before using the other four international samples. However, the fit indices did not support a good model (Singapore fathers—χ^2^/df = 4.04, CFI = 0.826, TLI = 0.813, RMSEA = 0.072; Singapore mothers—χ^2^/df = 4.11, CFI = 0.835, TLI = 0.823, RMSEA = 0.070; Malaysia fathers—χ^2^/df = 1.83, CFI = 0.848, TLI = 0.837, RMSEA = 0.061; Malaysia mothers—χ^2^/df = 1.63, CFI = 0.888, TLI = 0.880, RMSEA = 0.052). The model was improved using recommendations proposed by Hair et al. [37] and Awang [38], where items with low loadings (<0.50) were removed, but each item was removed one at a time and the loading values of all items were inspected again because the removal of any item altered the loading values of other items. This process involved 12 separate steps in which 11 items were removed: RQRN40, RQRN11, RQRN50, RQRN10, RQRN5, RQRN30, RQRN25, RQRN29, RQRN44, RQRN16 and RQRN22. Upon inspection, another five items with loadings < 0.50 appeared, although four of these items had loadings very close to 0.50 (0.496, 0.497, 0.493 and 0.481). However, one item (RQRN62), which had a loading of 0.45 and was part of the Fear of Harm and Illness subscale, was removed. The removal of this item resulted in only two items in this subscale (RQRN 52, RQRN 61), and as a result, this entire subscale with three items was removed. This removal resulted in item RQRN 19 having a loading value of 0.48, which was also removed. Item RQRN 26 was also removed as recommended by Awang [38] because it had the highest modification indices. At this scale refining stage, 16 items were removed from the initial 57 items, leaving behind 10 subscales and 41 items and they were Degradation and Rejection, Competitiveness and Status Seeking, Emotional Inhibition and Deprivation, Over-Control, Undependability and Irresponsibility, Overprotection and Overindulgence, Insufficient Guidance and Neglect, Intrusiveness and Exploitation, Social Exclusion, and Punitiveness and Abuse. By this stage, all subscales, except for Overprotection and Overindulgence, and Undependability and Irresponsibility in two of the four samples, had values of AVE and loading values above 0.50. Given the clinical significance of this subscale, it was included in the final model. This factor model was, then, ready for CFA, MGCFA and psychometric testing, which was conducted in Study 2. However, if this resulted in subsequent CFA fit indices not being adequate, either one or both subscales would be removed. 

## 7. Study 2

### 7.1. Model Fit Assessment and Psychometric Testing of YPI-R3

#### 7.1.1. Samples

The four international samples (USA, *n* = 396; South Africa, *n* = 390; Nigeria, *n* = 364; India, *n* = 306) were used in Study 2 to assess model fit using CFA and MGCFA. The samples from Singapore and Malaysia used in Study 1 were also used for the psychometric testing of the final version of the YPI-R3 in Study 2. 

#### 7.1.2. Measures 

All the measures below were used to psychometrically validate the YPI-R3 and these were administered only to the samples from Singapore and Malaysia. The YPI-R3 and Young Schema Questionnaire (YSQ-S3) were administered to the four international samples.

- *The Mini-International Personality Item Pool (Mini-IPIP)*

The Mini-IPIP [42] consists of 20 items and measures the Big Five personality traits (Agreeableness, “Feel others’ emotions”; Conscientiousness, “Like order”; Extraversion, “Talk to a lot of different people at parties”; Intellectual Openness, “Have difficulty understanding abstract ideas”; and Neuroticism, “Get upset easily”). A Likert scale is used with scores that range from 1 (*Wildly inaccurate*) to a score of 5 (*Very accurate*). A high test–retest correlation in the short term (0.62 to 0.87) and long term (0.68 to 0.86) was shown by this scale [42]. As a demonstration of convergent validity, the YPI-R3 subscale was hypothesized to correlate negatively with the subscales of conscientiousness, but to correlate positively with traits such as neuroticism with low to moderate effect sizes (|*r*| = 0.10 to 0.50) [43]. Cronbach’s alpha reliability values ranged from 0.65 to 0.77 [42].

- *Depression, Anxiety and Stress Subscales (DASS-21)*

The DASS-21 [44] scale has 21 items; these measure three aspects of emotional distress: Depression, “I found it difficult to work up the initiative to do things”; Anxiety, “I experienced breathing difficulty”; and Stress, “I felt that I was using a lot of nervous energy”. A 4-point Likert scale was used, ranging from 0 (*Did not apply to me at all*) to 4 (*Applied to me very much or most of the time*). This instrument has high concurrent validity as measured by Antony et al. [44] with *r* > 0.50 with the Beck Depression Inventory and Beck Anxiety Inventory [45]. It was hypothesized that the YPI-R3 would show low to moderate effect sizes (|*r*| = 0.10 to 0.50) with the YPI-R3 for showing of convergent validity. Cronbach’s alpha reliability values were 0.94, 0.87 and 0.91 for the three subscales of Depression, Anxiety and Stress, respectively [44].

- *The Gratitude Questionnaire–6 (GQ-6)*

For the purposes of this study, the GQ-6 scale [46] was used as a more distal measure, and it has six items that measure the disposition to experience gratitude. It uses a Likert scale, ranging from 1 (*Strongly disagree*) to a score of 7 (*Strongly agree*). An item example is “I have so much in life to be thankful for”. The GQ-6 scale correlated significantly and negatively with several measures of impaired sleep quality (*r* = −0.11 to −0.29), positively with pre-sleep cognitions (*r* = 0.21) and other measures of well-being [47]. Cronbach’s alpha was reported to be from 0.76 to 0.84 [47]. It was hypothesized that the YPI-R3 would correlate negatively with GQ-6 to demonstrate convergent validity with low to moderate effect sizes (|*r*| = 0.10 to 0.50).

- *Satisfaction with Life Scale (SWLS)*

The SWLS [48] has five short items and is a more distal measure of life satisfaction. A seven-point Likert scale is used, ranging from 1 (*Strongly disagree*) to 7 (*Strongly agree*). Item example is “I am satisfied with my life”. According to Diener et al., the SWLS reported a two-month test–retest stability coefficient of 0.82 and a strong negative correlation with the Beck Depression Inventory [48,49]. The reliability of the Cronbach’s value was 0.87 [48]. It was expected for the YPI-R3 to show convergent validity from low to moderate negative correlations with effect sizes (|*r*| = 0.10 to 0.50) with this scale.

- *Humor Styles Questionnaire (HSQ)*

The HSQ consists of 32 items, and each item represents a certain type of humor [50]. It uses a seven-point Likert scale, ranging from 1 (*Totally disagree*) to 7 (*Totally agree*). The two positive subscales of humor are Affiliative and Self-Enhancing. The former involves using humor to help others to feel connected and be at ease (e.g., “I enjoy making people laugh”), but the latter involves using humor when experiencing stress and challenges (e.g., “Even when I’m by myself, I am often amused by the absurdities of life”). The two negative types of humor are named Aggressive and Self-Defeating. The former involves putting people down or belittling them (e.g., “If someone makes a mistake, I will often tease them about it”), but the latter is about making fun of oneself (e.g., “I let people laugh at me or make fun at my expense more than I should”). The HSQ scale has been associated with well-being and is a more distal measure of daily life functioning [50]. The reliability Cronbach’s value was from 0.77 to 0.81 [50]. It was hypothesized that the YPI-R3 would correlate positively the Affiliate and Self-Enhancing types of humor, but negatively with the Aggressive and Self-Defeating ones to demonstrate convergent validity with low to moderate effect sizes (|*r*| = 0.10 to 0.50).

- *Eating Loss of Control Scale (ELOCS)*

This instrument [51] consists of 18 items, each with 2 parts, and measures feelings, cognitions and behaviors associated with lack of control during an eating episode. Questions to assess the frequency of binge eating, such as “During the past 4 weeks, how many times did you…?” represents the number of times a person experienced an eating episode resulting from a lack-of-control-related feeling or behavior [51]. Regarding lack of control, the question “On average during these times, how much did you…?” is asked. A Likert scale that ranges from 0 (*Not at all*) to 10 (*Extremely or entirely*) is used for this scale. These questions capture the degree to which individuals feel or behave related to loss of control. The item scores are averaged to produce a total score where higher total scores indicate greater control loss. Cronbach’s alpha values were reported to be 0.90 for the frequency items and the loss of control scale [51]. The mean loss of control scores did not correlate significantly with age, education or BMI, and did not differ by gender [51]. The scores for the frequency did differ by gender where women reported higher scores than men. The ELOS correlated with the Eating Disorder Examination scales positively with the frequency of objective bulimic events showing convergent validity. It was expected for the YPI-R3 to correlate positively with both frequency as well as lack of control as a show of convergent validity.

- *YPI-R3*

The factor structure of the YPI-R3 that had emerged from Study 1 consisted of 10 factors and 41 items. This scale has the same 6-point Likert scale as YPI-R2 ranging from 1 (*Completely untrue)* to 6 (*Describes him/her perfectly)* that measures retrospectively perceptions of parenting experiences. For item examples see “Measures” for YPI-R2 under Study 1. It was expected that in Study 2 the YPI-R3 would also be a robust and cross-culturally acceptable model that also demonstrates good psychometric properties of validity and reliability.

- *YSQ-S3*

This instrument is the latest version that measures 18 negative schemas [52]. It uses a six-point Likert scale, which ranges from 1 (*Completely untrue of me*) to 6 (*Describes me perfectly*). Item examples are “I don’t have people to give me warmth, holding, and affection” (Emotional Deprivation schema) and “I find myself clinging to people I’m close to because I’m afraid they’ll leave me” (Abandonment schema). This instrument was validated in a Korean population [53], where positive correlations were shown between all 18 schemas and measures of depression and anxiety taken from the subscales of the Symptom Checklist (SCL-90-R, [54]). In this same study, the factorial structure of all 18 negative schemas was confirmed using CFA. A study in Germany, using the community and clinical samples [16], also validated the YSQ-S3. The reliability values of 17 subscales were > 0.70 (Entitlement schema was 0.67). Convergent validity with the SCL-K-9, which is a shorter version of the SCL-90-R [55], was shown with negative schemas. The 18 negative schemas (except Unrelenting Standards) also correlated positively with the personality disorder symptoms measured [56]. It was expected that the YPI-R3 would demonstrate convergent validity through positive correlations with the YSQ-S3 with low to moderate effect sizes *(*|*r*| = 0.10 to 0.50).

### 7.2. Procedure and Statistical Analyses for Study 2

After obtaining the factor structure of the YPI-R3 from Study, 1 the first aim of Study 2 was to confirm the factor structure on four other samples, namely USA, South Africa, Nigeria and India, each with separate rating for fathers and mothers, using CFA and multi-group CFA (MGCFA). Both single group CFA and multi-group CFA (MGCFA) were conducted using a weighted least-squares means and variance adjusted estimation algorithm due to the ordered-categorical nature of the response scales [57]. Fit indices of each hypothesized model for the CFA were measured using two absolute fit indices (the root-mean-square error of approximation, RMSEA < 0.05, and the normed chi-square [58,59]), the comparative fit index (CFI ≥ 0.95) and one non-normed fit index (Tucker–Lewis index, TLI ≥ 0.95). Various methods to assess the invariance of the factor structure [60] were used for these samples: (1) configural invariance, which means that the same factor structure was used across groups; (2) metric invariance, which means that same factor loadings were used across groups; (3) scalar invariance, which means that the same item intercepts were used across groups; (4) error invariance, which means that the same error variance was used across groups; (5) factor variance invariance, which means that same factor variance was used across groups; (6) factor covariance, which means that the same factor covariance was used across groups; and (7) factor mean invariance, which means that the same factor mean was used across groups. The reliability values were tested using Cronbach’s alpha (*α*) and omega (*ω*) values, and factors with values of *α* ≥ 0.65 for newly developed instruments were acceptable [61]. All values of *ω* should exceed the acceptable level of 0.70, as proposed by Fornell and Larcker [62]. 

The second and last aim of Study 2 was to subject the YPI-R3 to psychometric testing. Convergent validity was evaluated using the Singapore sample on the IPIP, DASS-21, GQ6, SWLS and HSQ. The associations with negative schemas measured by YSQ-S3 were performed using the other four samples, USA, South Africa, Nigeria and India, each with separate ratings of fathers and mothers. If all six subscales of the YPI-R2 were retained in the YPI-R3, it was determined a priori that YPI-R3 would possess comparable construct validity as the YPI-R2 as demonstrated by Louis et al. [15] (to limit the number of questionnaire used measures of other established parenting patterns namely the s-EMBU, CTQ and PARQ were not included in the battery of instruments for the Singapore and Malaysia samples). The guidelines used to assess effect sizes were as follows: small, *r* = 0.1; medium, *r* = 0.30; and large, *r* = 0.50 [63]. Finally, hierarchical multiple regression was used to see if the YPI-R3 would predict psychopathology, personality traits, emotional distress, negative schemas and other distal functioning measures, such as life satisfaction, gratitude and humor styles, after controlling for age and gender. The predictor variables were entered using the following two steps: (1) age and gender; and (2) all 10 subscales from the YPI-R3. 

## 8. Results for Study 2

### 8.1. Model Fit Assessment Using CFA and MGCFA 

Having refined the YPI-R3 by removing less robust items, the model was now subjected to CFA and MGCFA. CFA results with the inclusion of the Overprotection and Overindulgence and the Undependability and Irresponsibility subscales showed adequate-good fit indices for the ratings of fathers and mothers on all four international samples—USA, South Africa, Nigeria and India (see Table 2). MGCFA showed that invariance was obtained on all seven levels for the fathers’ and mothers’ samples as shown in Table 3 and Table 4, respectively. These important findings from both CFA and MGCFA on four other international samples showed that the YPI-R3 scale is a robust, consistent and acceptable across various cultures. Comparison between the results of MGCFA and CFA for the YPI-R2 [15] and YPI-R3 from this study revealed the following: The MGCFA results for the YPI-R3 were far better than those for YPI-R2 [15], where for the latter, invariance was only achieved only at two levels for both fathers’ and mothers’ samples. Single group CFA indices from this study for the YPI-R3 were also higher, providing support that the YPI-R3 model again was an improvement to the YPI-R2. When these two scales were compared, the six subscales of the YPI-R2 were replicated in the YPI-R3 with the addition of four other subscales labeled as Neglect and Insufficient Guidance, Intrusiveness and Exploitation, Social Exclusion, and Undependability and Irresponsibility. Moreover, the Over-Control subscale appeared in the fathers’ and mothers’ samples for the YPI-R3 in this study, whereas it only appeared in the mothers’ sample in the YPI-R2 [15]. Therefore, three out of the four subscales that were previously rejected became robust in this study. One new subscale emerged, which was Neglect and Insufficient Guidance. Good reliability values represented by *α* and *ω* values (see Table 5) were obtained for each of the 10 subscales with 41 items of the YPI-R3 scale. A more balanced representation for the Overprotection and Overindulgence subscale was also achieved so that there were two items representing the Overprotection dimension and two for Overindulgence. 

### 8.2. Psychometric Testing of the YPI-R3

The YPI-R2 scale demonstrated construct validity as it correlated significantly with the subscales of other established measures of past parenting patterns, namely the s-EMBU, the CTQ and the PARQ, with medium effect sizes of 0.30, 0.29 and 0.42, respectively. Since the YPI-R3 retained all six subscales of YPI-R2, it was determined a priori that the YPI-R3 subscales would also follow suit and demonstrate significant associations with these established past parenting measures. The following measures were administered to test for convergent validity using the Singapore sample: the IPIP, Gratitude scale, DASS-21, YSQ (negative schemas), SWLS, Humor and ELOCS. Of particular interest were the performances of the four new subscales of the YPI-R3, namely, Undependability and Irresponsibility, Neglect and Insufficient Guidance, Intrusiveness and Exploitation, and Social Exclusion.

Highly significant correlations (*p* < 0.01) were found between the 4 new subscales of the YPI-R3 and the 15 subscales/scales of the IPIP, Gratitude, DASS-21, SWLS, Humor and ELOCS scales for ratings of fathers and mothers (see Table 6 and Table 7). The average statistically significant correlations values (|*r*|) in the fathers’ sample between the YPI-R3 scales and measures that were administered were as follows: IPIP = 0.105; Gratitude scale = 0.110; DASS-21 = 0.127; SWLS = 0.108; Humor = 0.129; and ELOCS = 0.129. The values (|*r*|) for the mothers’ sample were: IPIP = 0.118; DASS-21 = 0.152; SWLS = 0.131; Humor = 0.162; and ELOCS = 0.121. While these effect sizes were significant, they were small [63]. However, studies have shown that the effect sizes demonstrated by other established measures of past parenting patterns were in the same range. For example, the correlations between s-EMBU with neuroticism, extraversion and self-esteem were 0.20, 0.19 and 0.22, respectively. Further, small effect sizes (|*r*| = 0.26 and 0.22) also emerged between s-EMBU with measures of personality disorder symptoms and depression [64]. Similar effect sizes also appeared between PARQ, which measures past parenting patterns (|*r*| = 0.06 to 0.14) and child adjustment measures [65]. Therefore, the measures of past parenting patterns tend to yield small, but statistically significant, correlations with other measures of well-being, emotional distress and negative schemas, which was consistent with the results from this study.

For fathers’ (see Supplementary Materials, Tables S4–S7) and mothers’ (see Supplementary Materials, Tables S8–S11) samples from the four countries—the United States, South Africa, Nigeria and India—a medium effect size (*p* < 0.01) and positive associations were observed between the YPI-R3 and many of the negative schemas. The subscales that yielded the largest effect sizes were Overprotective and Overindulgent, Neglect and Insufficient Guidance, Intrusiveness and Exploitation, Social Exclusion, Undependability and Irresponsibility, and Degradation and Rejection. Four of these six subscales were newer ones of the YPI-R3. The associations with negative schemas further support the convergent validity of the YPI-R3 scale. 

Hierarchical multiple regression showed that the YPI-R3 was able to generate prediction equations that were statistically significant (*p* < 0.001) in 13 out of 15 dependent variables found in the IPIP, Gratitude scale, DASS-21, SWLS, Humor and ELOCS scales in the fathers’ sample. The YPI-R3 also generated the same with 10 (8 with *p* < 0.001, 2 with *p* < 0.01) out of the 15 dependent variables in the mothers’ sample. This was over and above the variance contributed by age and gender (see Table 8 and Table 9). The YPI-R3, therefore, has an impressive predictive capability for the measures of personality traits (IPIP), emotional distress (DASS-21), psychopathology (ELOS) and other distal measures, such as satisfaction with life (SWLS), gratitude and humor. 

## 9. Discussion

Baumrind’s [2] and Maccoby and Martin’s [3] three broad maladaptive parenting constructs—Authoritarian, Permissive and Neglectful—have proven valuable, but are limited in their range as are other established measures of past parenting patterns in that virtually all contain only two to three maladaptive parenting patterns. More deviant parenting patterns need to be identified since these have been shown to be linked with personality disorders [7,9,10,11]. The aim of this study was to develop and psychometrically validate a comprehensive measure of maladaptive parenting patterns that would help parents, clinicians and educators to identify the nature of these patterns and its link to psychopathology and provide a basis for further research into the nature and impact of these constructs. 

The original YPI, developed by Young et al. [7] from the vantage point of schema therapy, was used as a starting point. This scale hypothesized 17 negative parenting patterns paralleling the 17 negative schemas. This measure added breadth and specificity to the assessment of patterns, but was never psychometrically validated. Louis et al.’s [15] work resulted in an improved and psychometrically validated version of the YPI, known as YPI-R2, which consists of six subscales [15]. However, during its development, four subscales measuring other deviant parenting patterns were rejected, likely because their items were not sufficiently robust. This study was set out to develop an improved version of the YPI-R2 by developing new items that represented the four rejected subscales as well as improving some of its existing ones, thereby resulting in a scale known as the YPI-R3 that would measure a fuller range of maladaptive parenting patterns than what is currently available in the literature. 

This study was split into two parts. In Study 1, a robust model of the YPI-R3 emerged, which consisted of 10 subscales (Degradation and Rejection, Competitiveness and Status Seeking, Emotional Inhibition and Deprivation, Over-Control, Undependability and Irresponsibility, Overprotection and Overindulgence, Insufficient Guidance and Neglect, Intrusiveness and Exploitation, Social Exclusion, and Punitiveness and Abuse), and 41 items. This was achieved after subjecting an initial pool of 74 items to rigorous analyses using samples from Singapore and Malaysia with separate ratings for fathers and mothers. 

Study 2 was designed to test the YPI-R3 cross-culturally, using four international samples (USA, South Africa, Nigeria and India). Finally, it was subjected to psychometric testing by comparing it to other established instruments using all six international samples. Statistically significant differences in groups existed among men and women in sample sizes and age, but not in races. The greater number of women was likely due to the incentives being centered on parenting. Cross-cultural support was demonstrated using CFA and MGCFA where the YPI-R3 generated good CFA fit indices and achieved invariance at all seven levels of testing. The YPI-R3 was, therefore, shown to be stable across Western and Asian cultures. 

The YPI-R3 was also an improvement on the YPI-R2 in following areas. The fit indices for the YPI-R3 model obtained from this study were better for both single-group CFA and MGCFA. Specifically, the MGCFA invariance for the YPI-R2 was only obtained at two levels for both the fathers’ and mothers’ samples, but for the YPI-R3, all seven levels were achieved showing that it was more robust cross-culturally. The YPI-R2 scale contained many more items than deemed necessary for some of its subscales. For example, there were eight items in the Degradation and Rejection and the Overprotection and Overindulgence subscales. In the revised and improved YPI-R3 scale, the number of items for some of these subscales were reduced, which resulted in the YPI-R3 containing 10 subscales with 41 items in comparison to the YPI-R2 with 6 subscales and 36 items. Therefore, with an increase of five items in the former, there was an increase of four subscales, rendering the YPI-R3 more comprehensive as a scale. The Overprotection and Overindulgence subscales in the YPI-R3 now contain two items for Overprotection and two for Overindulgence, which is better balanced than the arrangement of six items for Overprotection and two items for Overindulgence found in the YPI-R2. In summary, the YPI-R3 was a significant improvement to the YPI-R2 scale, which, in turn, was a significant improvement on the original YPI. The original YPI had 72 items and only 16 of these ended up as viable in the YPI-R2. Only 13 of the original 72 items ended up in the YPI-R3. Therefore, the vast majority of the items in the YPI-R3 were new items that are more robust than the original ones in the YPI. 

The psychometric soundness of the YPI-R3 was demonstrated in the following. In line with the core tenet of schema therapy, statistically significant correlations were obtained between all subscales of the YPI-R3 and the 18 negative schemas in all the 4 international samples [7,15]. However, there was not a clear pattern, as hypothesized by Young et al. (2003) [7], where a specific type of maladaptive parenting pattern resulted in the development of a specific negative schema (i.e., that 17 subscales of the YPI have strong correlations with the negative schema they were derived from). Rather, each negative schema had significant negative associations with many of the scales making up the YPI-R3 in all international samples. Furthermore, Young et al. [7] also hypothesized that the negative schema of Social Isolation was due to problems in relationships with one’s peer group rather than family, but results from this study showed that a number of the YPI-R3 subscales had significant associations with the negative schema of Social Isolation. It is also noteworthy that the most significant associations between negative schemas and the subscales of YPI-R3 were with Overprotective and Overindulgent, Neglect and Insufficient Guidance, Intrusiveness and Exploitation, Social Exclusion, Undependability and Irresponsibility, and Degradation and Rejection scales. Of these six subscales in the YPI-R3, four are the newer ones that emerged in this study. This underscores the significance of these newer subscales.

Significant correlations between the YPI-R3 and measures of psychopathology (ELOCS), personality traits (Mini-IPIP), emotional distress (DASS-21 for depression, anxiety and stress) and other distal measures, such as satisfaction with life (SWLS), gratitude and humor, demonstrated convergent validity. Hierarchical regression also showed good predictive capability for the YPI-R3, with these measures resulting in statistically significant variance in 12 out of 15 dependent variables for the fathers’ sample and 10 out of 15 for the mothers’ sample. In conclusion, the YPI-R3, with its 10 subscales, shows potential for its use in both clinical and research work and is a reliable scale for measuring a fuller range of past maladaptive parenting patterns, both deviant and normal. This overcomes a clear limitation that exists in the current literature for measuring past parenting patterns. 

## 10. Limitations

There were several limitations in this study. The first was that incentives to attend a workshop on parenting may have been the reason why significantly more women than men signed up to take part in the study. This sample bias could limit the generalizability of the findings. Second, the survey administered did not take into account participants’ gender identity and sexual orientation. Regarding gender identity, the level of social acceptance on matters such as rights of gay marriages and homosexuality is very low especially in Asian countries such as Malaysia and Singapore [66], and to lesser extent India and South Africa [67]. This may likely be linked to the fact that such acts are still considered illegal in Nigeria, Malaysia and Singapore [68]. Public opinion is a serious consideration in such collectivist countries that tend to not promote self-expression as much as individualistic cultures [14]. As a result, participants in collectivist cultures will likely be more resistant to answering questions regarding gender identity in a survey. Therefore, this classification was not asked in any of the samples in order to maintain consistency in all the questionnaires for all six samples. In addition, again due to social courtesy in collectivist countries [14], participants were also not asked about their sexual orientation. Moreover, it was not part of the hypotheses of this study—the reason being there is currently no good consistent scientific evidence to suggest that early parent–child interactions have a primary causal impact on sexual orientation in adolescents or adults [69]. However, this was a limitation, and future studies in countries where such classification is not illegal should further explore these complex issues. Third, there were questions about negative schemas and past parenting experiences that may have been challenging for some participants who may not have been able to recollect and assess themselves accurately. The fourth limitation was that the data were cross-sectional and non-experimental and so no causal conclusions could be drawn.

## 11. Implications and Future Studies

Baumrind’s parenting constructs were developed from qualitative observations of normal parent–child interactions. This model did not include dimensions arising from deviant parenting practices, such as abuse and neglect [5,6]. Other established instruments are also limited in the range of their constructs, measuring only two to three past negative parenting patterns. The YPI-R3, with its 10 subscales, will allow clinicians to more fully assess maladaptive past parenting patterns. This can help to further our understanding of the role of parenting across a range of developmental outcomes, including the development of personality disorders [7,9,10,11] to which they have been linked. Since the meaning of deviant parenting is influenced by culture, the YPI-R3 is able to tap into the facets of maladaptive parenting that are both normative within a given culture as well as deviant. Drawing from Baumrind’s model [3], the former can be measured by the subscales of Emotional Inhibition and Deprivation and Controlling, which reflect the dimensions of warmth and control, respectively. Other normal practices of parenting that are maladaptive are represented by Degradation and Rejection, Overprotection and Overindulgence, and Competitiveness and Status Seeking, provided such patterns are not taken to extremes. However, the YPI-R3 can now measure more traumatic, abusive and neglectful parenting patterns represented by the subscales of Intrusiveness and Exploitation, Punitiveness and Abuse, Social Exclusion, Undependability and Irresponsibility, and Neglect and Insufficient Guidance. These new more nuanced set of constructs hold the promise of shedding more light on investigations into the ways in which past maladaptive parenting has contributed to negative developmental outcomes. For example, Rejection can be registered in at least two major ways, through being degraded (the Degradation and Rejection subscale) or through punitiveness or abuse (the Punitiveness and Abuse subscale), and both can now be measured by the YPI-R3 scale, which takes this distinction into account. As a result, clinicians and parents can gain a fuller understanding of the origin of such deviant parenting patterns and perhaps link the development of specific personality disorders in adulthood with specific parenting patterns in childhood. For example, the schema therapy model postulates that the development of narcissism has its roots in conditional love conveyed to a child during his or her early years. It is believed that individuals with this disorder frequently had to earn the love of their parents’ affection by performing in a manner that would gain their approval. These kind of early childhood experiences can be assessed by some of the subscales of the YPI-R3, such as Competitiveness and Status Seeking, Emotional Inhibition and Deprivation, Punitiveness and Abuse, and Degradation and Rejection. If a measure of past parenting patterns has only two or three subscales, it would be hard to pinpoint the specific nature of their early parenting experiences. Typically, only one subscale related to this theme, for example, a measure of rejection, would be available. However, this would miss a potential blending of distinct elements critical for a fuller understanding of the likely origins of this pathology. This would also assist a therapist in the fuller exploration of these specific experiences. Individuals diagnosed with Borderline Personality Disorder typically have early family environments that were unsafe, toxic and unstable, and often experienced severe abuse, both emotional, physical and sexual, as well as deprivation of nurturance. In addition, they were frequently abandoned. Their early parenting experiences are therefore manifested in a wide range of maladaptive parenting patterns that are likely to be detected by most of the 10 subscales of the YPI-R3. Measures that assess only two or three maladaptive parenting patterns will no doubt detect some of these experiences, but will fall short of facilitating a full understanding of the links between the early family environment and this disorder. Furthermore, a more comprehensive measure of past parenting experiences can be helpful for clients who have difficulty recollecting past unpleasant childhood experiences since the concrete examples contained in the items making up the scales often help to recognize patterns that they are unable to recall independently. Alternatively, clients with narcissistic tendencies often deny personal failings and vulnerabilities, but are often comfortable reporting on negative experiences with their parents. This then serves as an important entry point to the personal vulnerabilities. The breadth of the measure will allow for a fuller counter to these two types of avoidance. 

Another important implication is that parenting patterns that are viewed as culturally normative may be shown, using the YPI-R3, to result in unfavorable outcomes in children. For example, Japanese mothers tend to promote less expression in order to promote emotional maturity, self-control, social courtesy and interdependence [70]. Taken to an extreme, such a pattern may take the form of the YPI-R3 construct Emotional Inhibition and Deprivation. By contrast, American mothers try to promote autonomy, assertiveness, verbal competence and self-actualization [70]. When this is overdone, this may take the form of the YPI-R3 construct Overprotection and Overindulgence. Therefore, culturally influenced parenting patterns that are viewed as normative can, when taken to extremes, have negative developmental outcomes as shown in this study. Therefore, it will be helpful for parents and educators to not jump to the conclusion that normative cultural parenting patterns are necessarily healthy. Child and adult well-being will be best served if cultural norms are viewed through the lens of empirical findings on developmental outcomes. Another important implication is that this study has shown that maladaptive parenting patterns originating from fathers can be as dysfunctional as mothers given the similar effect sizes as measured by a wide array of instruments in this study. This underscores the importance of fathers in child rearing. It also showed that the quality of parenting was not necessarily associated with a specific gender of the parent. A final implication is that it will be beneficial for clinicians and psychologists to combine the use of this scale with other psychological tests due to its significant level of predictive capability. 

Since all samples were drawn from a non-clinical, predominantly English-speaking community, future studies should examine the replicability of these results on non-English speaking communities and in clinical populations. Future studies should also focus on testing the YPI-R3 across gender (men/women) and parenting (father/mother) and investigate the invariance at all seven levels using multi-cultural samples. Future studies can also be conducted to see if different parenting patterns affect different stages of childhood development. Potential differences in parenting patterns could be explored in different cultures, countries and socioeconomic groups to see to what extent there are “universal” parenting patterns that apply across all groups. Future studies could also focus on the effects of the various parenting patterns of the YPI-R3 on different childhood temperaments. Studies could also be conducted to see if certain parenting patterns are more prone to giving rise to a certain personality disorder. Finally, it is reasonable to assume that psychological instruments evolve and that further testing will demonstrate that this measure can be reduced to make it more efficient by expanding to include new constructs or important nuances within the current patterns. Some instruments have a more succinct version, such as the Mini IPIP, as opposed to the longer IPIP [42]. Another example is the short version of the YSQ (the YSQ-S3 with 90 items) and the long version (the YSQ-L3 with 232 items) [7]. Future studies can also be conducted on the YPI-R3 using Rasch analysis [71], which is a psychometric technique that can be used to improve the precision of this instrument. This method of analysis will also create alternative forms of measurement. 

## Figures and Tables

**Table 1:** Demographic characteristics of the samples.

Characteristics	Categories	USA	South Africa	Nigeria	India	Singapore	Malaysia
**Gender**	Men	147	159	209	169	260	83
	Women	249	231	155	137	371	149
	Total	396	390	364	306	631	232
**Age**	Mean	43.69	42.11	45.7	42.39	46.22	41.40
	SD	9.12	6.79	7.19	7.67	22.34	17.40
**Missing**	>10%	0	0	0	0	3	3
**Race**	Chinese	N. A.	N. A.	N. A.	N. A.	508	205
	Indonesian	N. A.	N. A.	N. A.	N. A.	5	5
	Indian	N. A.	7	N. A.	N. A.	15	3
	Filipino	N. A.	N. A.	N. A.	N. A.	91	9
	Caucasian/White	104	65	N. A.	N. A.	2	2
	Black	52	135	N. A.	N. A.	N. A.	N. A.
	Latino	121	N. A.	N. A.	N. A.	N. A.	N. A.
	Asian	99	N. A.	N. A.	N. A.	N. A.	N. A.
	Coloured	N. A.	17	N. A.	N. A.	N. A.	N. A.
	Yoruba	N. A.	N. A.	191	N. A.	N. A.	N. A.
	Ibo	N. A.	N. A.	72	N. A.	N. A.	N. A.
	Hausa	N. A.	N. A.	5	N. A.	N. A.	N. A.
	North India	N. A.	N. A.	N. A.	31	N. A.	N. A.
	East India	N. A.	N. A.	N. A.	44	N. A.	N. A.
	South India	N. A.	N. A.	N. A.	138	N. A.	N. A.
	West India	N. A.	N. A.	N. A.	45	N. A.	N. A.
	Others	20	7	96	48	9	8
	Did not specify	0	159	0	0	1	0
	Missing	0	0	0	0	3	3
	Sample Size	396	390	364	306	628	229
	* Final Fathers Sample Size, *n*	259	318	328	277	592	222
	* Final Mothers Sample Size, *n*	281	372	344	289	628	229

* Final Fathers sample removed participants from the sample who did not grow up with a father. * Final Mothers sample removed participants from the sample who did not grow up with a mother.

**Table 2:** CFA fit indices with separate ratings for fathers and mothers from the USA (*n* = 259, 281), South Africa (*n* = 318, 372), Nigeria (*n* = 328, 344) and India (*n* = 277, 289) samples.

Model	Number of Parameters	*χ^2^*	*df*	*p*	*χ^2^/df*	CFI	TLI	RMSEA
**Mothers**								
**USA (*n* = 281)**	291	1481.13	734	<0.001	2.02	0.920	0.911	0.060 [0.056 0.065]
**South Africa (*n* = 372)**	291	1730.78	734	<0.001	2.36	0.949	0.942	0.060 [0.057 0.064]
**Nigeria (*n* = 344)**	291	1839.44	734	<0.001	2.51	0.928	0.919	0.066 [0.062 0.070]
**India (*n* = 289)**	291	1562.01	734	<0.001	2.13	0.921	0.912	0.062 [0.058 0.067]
**Fathers**								
**USA (*n* = 259)**	291	1563.86	734	<0.001	2.13	0.922	0.913	0.066 [0.062 0.071]
**South Africa (*n* = 318)**	291	1761.54	734	<0.001	2.40	0.942	0.936	0.066 [0.062 0.070]
**Nigeria (*n* = 328)**	291	1557.37	734	<0.001	2.12	0.952	0.946	0.058 [0.054 0.063]
**India (*n* = 277)**	291	1639.05	734	<0.001	2.23	0.911	0.900	0.067 [0.062 0.071]

**Table 3:** MGCFA using fathers’ samples from the USA (*n* = 259), South Africa (*n* = 318), Nigeria (*n* = 328) and India (*n* = 277).

Model	Number of Parameters	*χ2*	*df*	*p*	*χ2/df*	CFI	TLI	RMSEA	Comparison	Decision
**Configural invariance**	1156	6565.03	2944	<0.001	2.229969	0.935	0.928	0.065 [0.062 0.067]	-	Accept
**Metric invariance**	1066	6773.402	3034	<0.001	2.232499	0.933	0.928	0.065 [0.063 0.067]		
		−356.491	−90	(<0.001)		(0.002)	(<0.001)	(<0.001)	Configural vs. Metric	Accept
**Scalar invariance**	609	7389.172	3491	<0.001	2.116635	0.930	0.935	0.061 [0.060 0.063]		
		−1105.5	−457	(<0.001)		(0.003)	(−0.007)	(−0.004)	Metric vs. Scalar	Accept
**Residual variance invariance**	486	7280.346	3614	<0.001	2.014484	0.935	0.941	0.059 [0.057 0.061]		
		−370.087	−123	(<0.001)		(−0.005)	(−0.006)	(−0.002)	Scalar vs. Residual	Accept
**Factor variance invariance**	456	7297.206	3644	<0.001	2.002526	0.935	0.941	0.058 [0.056 0.060]		
		−130.106	−30	(<0.001)		(<0.001)	(<0.001)	(−0.001)	Residual vs. Factor variance	Accept
**Factor covariance invariance**	324	6340.983	3776	<0.001	1.679286	0.954	0.96	0.048 [0.046 0.050]		
		−317.873	−132	(<0.001)		(−0.019)	(−0.019)	(−0.010)	Factor variance vs. Factor covariance	Accept
**Factor mean invariance**	294	6758.992	3806	<0.001	1.775878	0.947	0.955	0.051 [0.049 0.053]		
		−200.422	−30	(<0.001)		(0.007)	(0.005)	(0.003)	Factor covariance vs. Factor mean	Accept
**Acceptance criteria for indices (differences)**						>0.9	>0.9	<0.06		
						(<0.01)	(<0.01)	(<0.015)		

**Table 4:** MGCFA using mothers’ samples from the USA (*n* = 281), South Africa (*n* = 372), Nigeria (*n* = 344) and India (*n* = 289).

Model	Number of Parameters	*χ2*	*df*	*p*	*χ2/df*	CFI	TLI	RMSEA	Comparison	Decision
**Configural invariance**	1156	6702.769	2944	<0.001	2.276756	0.93	0.922	0.063 [0.061 0.065]	-	Accept
**Metric invariance**	1066	6923.653	3034	<0.001	2.282021	0.927	0.921	0.063 [0.061 0.065]	Configural vs. Metric	Accept
		−376.075	−90	(<0.001)		(0.003)	(0.001)	(<0.001)		
**Scalar invariance**	609	7302.121	3491	<0.001	2.091699	0.929	0.933	0.058 [0.056 0.060]	Metric vs. Scalar	Accept
		−874.001	−457	(<0.001)		(−0.002)	(−0.012)	(−0.005)		
**Residual variance invariance**	486	7344.233	3614	<0.001	2.032162	0.930	0.937	0.057 [0.055 0.059]	Scalar vs. Residual	Accept
		−435.011	−123	(<0.001)		(−0.001)	(−0.004)	(−0.001)		
**Factor variance invariance**	456	7111.97	3644	<0.001	1.951693	0.935	0.942	0.054 [0.053 0.056]	Residual vs. Factor variance	Accept
		−62.504	−30	−0.0005		(−0.005)	(−0.005)	(−0.003)		
**Factor covariance invariance**	324	5741.454	3776	<0.001	1.520512	0.963	0.968	0.040 [0.038 0.042]	Factor variance vs. Factor covariance	Accept
		−228.597	−132	(<0.001)		(−0.028)	(−0.026)	(−0.014)		
**Factor mean invariance**	294	6229.461	3806	<0.001	1.636748	0.955	0.961	0.045 [0.043 0.046]	Factor covariance vs Factor mean	Accept
		−224.295	−30	(<0.001)		(0.008)	(0.007)	(0.005)		
**Acceptance criteria for indices (differences)**						>0.9	>0.9	<0.06		
						(<0.01)	(<0.01)	(<0.015)		

**Table 5:** Reliability values of *α* and *ω* of YPI-R3 subscales using samples with ratings for fathers and mothers from Singapore (*n* = 592, 628) and Malaysia (*n* = 222, 229).

	Mothers				Fathers			
	Kuala-Lumpur		Singapore		Kuala-Lumpur		Singapore	
	*α*	*ω*	*α*	*ω*	*α*	*ω*	*α*	*ω*
**OC**	0.87	0.91	0.89	0.91	0.88	0.90	0.88	0.91
**EID**	0.79	0.85	0.80	0.86	0.77	0.83	0.81	0.86
**UI**	0.78	0.77	0.66	0.77	0.76	0.84	0.73	0.82
**OO**	0.71	0.80	0.73	0.80	0.68	0.75	0.68	0.77
**NIG**	0.67	0.74	0.70	0.74	0.70	0.73	0.72	0.77
**CSS**	0.81	0.85	0.80	0.85	0.80	0.85	0.79	0.85
**IE**	0.76	0.80	0.67	0.80	0.69	0.82	0.67	0.81
**DR**	0.81	0.90	0.87	0.90	0.84	0.88	0.88	0.91
**SE**	0.76	0.75	0.68	0.75	0.70	0.77	0.70	0.77
**PA**	0.77	0.85	0.79	0.86	0.79	0.85	0.79	0.85

OC = Over-Control; EID = Emotional Inhibition and Deprivation; UI = Undependability and Irresponsibility; OO = Overprotection and Overindulgence; NIG = Neglect and Insufficient Guidance; CSS = Competitiveness and Status Seeking; IE = Intrusiveness and Exploitation; DR = Degradation and Rejection; SE = Social Exclusion; PA = Punitiveness and Abuse.

**Table 6:** Correlations between the YPI-R3 and IPIP, Gratitude, DASS-21, SWLS and Humor scales using the Singapore fathers’ sample (*n* = 592).

	OC	EID	UI	OO	NIG	CSS	IE	DR	SE	PA
**IPIP Agreeableness**	−0.023	−0.135 **	−0.100 *	−0.067	−0.083 *	−0.028	−0.114 **	−0.094 *	−0.185 **	−0.010
**IPIP Conscientiousness**	−0.123 **	0.042	−0.139 **	−0.182 **	−0.005	−0.066	−0.139 **	−0.135 **	−0.058	−0.102 *
**IPIP Extraversion**	−0.045	−0.206 **	0.004	−0.001	−0.114 **	0.031	−0.002	−0.098 *	−0.150 **	−0.033
**IPIP Intellect**	0.083 *	−0.166 **	−0.031	−0.051	−0.101 *	0.116 **	0.005	0.029	−0.033	0.109 **
**IPIP Neuroticism**	0.123 **	0.086*	−0.006	0.160 **	0.030	0.086*	0.086 *	0.184 **	0.121 **	0.103 *
**Gratitude**	0.011	−0.079	−0.103 *	−0.076	−0.058	−0.015	−0.130 **	−0.068	−0.096 *	−0.006
**DASS-21 Depression**	0.120 **	0.061	0.075	0.130 **	0.101 *	0.069	0.184 **	0.185 **	0.126 **	0.104 *
**DASS-21 Anxiety**	0.140 **	0.002	0.031	0.134 **	0.011	0.092 *	0.195 **	0.091 *	0.095 *	0.095 *
**DASS-21 Stress**	0.124 **	0.064	0.036	0.169 **	0.052	0.110 **	0.086 *	0.132 **	0.160 **	0.100 *
**SWLS**	−0.047	−0.038	−0.093 *	−0.107 **	−0.121 **	0.000	−0.083 *	−0.138 **	−0.069	−0.067
**Humor Affiliative**	0.018	−0.152 **	−0.042	−0.022	−0.071	0.060	−0.037	−0.010	−0.112 **	−0.011
**Humor Aggressive**	0.092 *	−0.006	0.127 **	0.187 **	−0.005	0.107 **	0.066	0.067	0.052	0.031
**Humor Self-Defeating**	0.208 **	0.057	0.106 **	0.285 **	0.029	0.200 **	0.138 **	0.184 **	0.125 **	0.114 **
**Humor Self-Enhancing**	0.025	−0.086 *	0.000	0.031	−0.043	0.071	−0.012	−0.012	−0.060	0.008
**ELOCS**	0.182 **	0.045	0.099 *	0.168 **	0.016	0.175 **	0.130 **	0.125 **	0.078	0.136 **

** Correlation is significant at 0.01 level; * Correlation is significant at 0.05 level. OC = Over-Control; EID = Emotional Inhibition and Deprivation; UI = Undependability and Irresponsibility; OO = Overprotection and Overindulgence; NIG = Neglect and Insufficient Guidance; CSS = Competitiveness and Status Seeking; IE = Intrusiveness and Exploitation; DR = Degradation and Rejection; SE = Social Exclusion; PA = Punitiveness and Abuse; SWLS = Satisfaction with Life Scale; ELOCS = Eating Loss of Control Scale; IPIP = International Personality Item Pool.

**Table 7:** Correlations between the YPI-R3 and IPIP, Gratitude, DASS-21, SWLS and Humor scales using the Singapore mothers’ sample (*n* = 628).

	OC	EID	UI	OO	NIG	CSS	IE	DR	SE	PA
**IPIP Agreeableness**	0.126 **	0.105 **	0.086 *	0.071	0.108 **	0.033	0.170 **	0.126 **	0.135 **	0.123 **
**IPIP Conscientiousness**	0.109 **	0.082 *	0.051	0.160 **	0.011	0.052	0.047	0.053	0.147 **	0.044
**IPIP Extraversion**	0.019	0.078	0.072	0.101 *	0.103 **	0.042	0.098 *	0.040	0.089 *	0.042
**IPIP Intellect**	0.071	0.180 **	0.077	0.116 **	0.090 *	−0.015	0.022	0.083 *	0.085 *	0.048
**IPIP Neuroticism**	0.070	0.046	0.075	0.143 **	0.071	0.001	0.043	0.045	0.125 **	0.058
**Gratitude**	−0.022	−0.042	0.016	0.050	−0.022	−0.026	−0.023	−0.041	−0.029	−0.038
**DASS-21 Depression**	0.166 **	0.066	0.050	0.158 **	0.076	0.072	0.169 **	0.207 **	0.168 **	0.137 **
**DASS-21 Anxiety**	0.129 **	−0.023	0.021	0.147 **	0.023	0.051	0.140 **	0.082 *	0.125 **	0.078
**DASS-21 Stress**	0.184 **	0.064	−0.013	0.231 **	0.010	0.129 **	0.069	0.165 **	0.150 **	0.130 **
**SWLS**	−0.149 **	−0.095 *	−0.114 **	−0.132 **	−0.083 *	−0.070	−0.127 **	−0.203 **	−0.122 **	−0.150 **
**Humor Affiliative**	0.025	0.113 **	−0.021	0.113 **	0.042	−0.010	0.050	0.046	0.101 *	0.016
**Humor Aggressive**	−0.001	0.053	0.008	0.050	0.041	0.057	0.001	0.002	−0.028	−0.028
**Humor Self-Defeating**	0.146 **	0.022	0.086 *	0.240 **	0.040	0.125 **	0.149 **	0.111 **	0.127 **	0.061
**Humor Self-Enhancing**	0.033	−0.058	0.054	0.078	0.040	0.098 *	0.054	−0.022	−0.020	−0.002
**ELOCS**	0.149 **	0.013	0.085 *	0.195 **	0.001	0.143 **	0.099 *	0.114 **	0.106 **	0.122 **

** Correlation is significant at 0.01 level; * Correlation is significant at 0.05 level. OC = Over-Control; EID = Emotional Inhibition and Deprivation; UI = Undependability and Irresponsibility; OO = Overprotection and Overindulgence; NIG = Neglect and Insufficient Guidance; CSS = Competitiveness and Status Seeking; IE = Intrusiveness and Exploitation; DR = Degradation and Rejection; SE = Social Exclusion; PA = Punitiveness and Abuse; SWLS = Satisfaction with Life Scale; ELOCS = Eating Loss of Control Scale; IPIP = International Personality Item Pool.

**Table 8:** Hierarchical regression analysis of the YPI-R3 predicting the IPIP, Gratitude, DASS-21, SWLS and Humor scales using the Singapore fathers’ sample (*n* = 592).

	R^2^	∆R^2^	∆F
**IPIP Agreeableness**			
Step1: Age, gender	0.014	0.014	4.15 *
Step 2: YPI-R3 scales	0.079	0.065	4.01 ***
**IPIP Conscientiousness**			
Step1: Age, gender	0.043	0.043	12.98 ***
Step 2: YPI-R3 scales	0.112	0.069	4.46 ***
**IPIP Extraversion**			
Step1: Age, gender	0.001	0.001	0.29
Step 2: YPI-R3 scales	0.072	0.071	4.35 ***
**IPIPIntellect**			
Step1: Age, gender	0.037	0.037	11.27 ***
Step 2: YPI-R3 scales	0.119	0.081	5.27 ***
**IPIP Neuroticism**			
Step1: Age, gender	0.044	0.044	13.19 ***
Step 2: YPI-R3 scales	0.125	0.082	5.34 ***
**Gratitude_1**			
Step1: Age, gender	0.005	0.005	1.49
Step 2: YPI-R3 scales	0.058	0.053	3.19 ***
**DASS-21 Depression**			
Step1: Age, gender	0.047	0.047	14.43 ***
Step 2: YPI-R3 scales	0.120	0.072	4.69 ***
**DASS-21 Anxiety**			
Step1: Age, gender	0.035	0.035	10.57 ***
Step 2: YPI-R3 scales	0.091	0.056	3.52 ***
**DASS-21 Stress**			
Step1: Age, gender	0.036	0.036	10.78 ***
Step 2: YPI-R3 scales	0.094	0.058	3.66 ***
**SWLS**			
Step1: Age, gender	0.011	0.011	3.14 *
Step 2: YPI-R3 scales	0.062	0.051	3.11 ***
**Humor Affiliative**			
Step1: Age, gender	0.026	0.026	7.64 ***
Step 2: YPI-R3 scales	0.056	0.031	1.85
**Humor Aggressive**			
Step1: Age, gender	0.050	0.050	15.23 ***
Step 2: YPI-R3 scales	0.102	0.052	3.30 ***
**Humor Self-Defeating**			
Step1: Age, gender	0.053	0.053	16.21 ***
Step 2: YPI-R3 scales	0.168	0.115	7.92 ***
**Humor Self-Enhancing**			
Step1: Age, gender	0.008	0.008	2.22
Step 2: YPI-R3 scales	0.037	0.029	1.73
**ELOCS**			
Step1: Age, gender	0.028	0.028	8.35 ***
Step 2: YPI-R3 scales	0.085	0.057	3.57 ***

* *p* ≤ 0.05; ** *p* < 0.01; *** *p* < 0.001; IPIP = International Personality Item Pool; SWLS = Satisfaction with Life Scale; ELOCS = Eating Loss of Control Scale.

**Table 9:** Hierarchical regression analysis of the YPI-R3 predicting the IPIP, Gratitude, DASS-21, SWLS and Humor scales using the Singapore mothers’ sample (*n* = 628).

	R^2^	∆R^2^	∆F
**IPIP Agreeableness**			
Step1: Age, gender	0.001	0.001	0.23
Step 2: YPI-R3 scales	0.047	0.046	2.94 **
**IPIP Conscientiousness**			
Step1: Age, gender	0.007	0.007	2.21
Step 2: YPI-R3 scales	0.058	0.051	3.28 ***
**IPIP Extraversion**			
Step1: Age, gender	0.032	0.032	10.17 ***
Step 2: YPI-R3 scales	0.057	0.025	1.61
**IPIP Intellect**			
Step1: Age, gender	0.010	0.010	3.15 *
Step 2: YPI-R3 scales	0.069	0.059	3.87 ***
**IPIP Neuroticism**			
Step1: Age, gender	0.010	0.010	3.23 *
Step 2: YPI-R3 scales	0.054	0.044	2.82 **
**Gratitude**			
Step1: Age, gender	0.026	0.026	8.27 ***
Step 2: YPI-R3 scales	0.043	0.017	1.05
**DASS-21 Depression**			
Step1: Age, gender	0.043	0.043	13.85 ***
Step 2: YPI-R3 scales	0.127	0.084	5.82 ***
**DASS-21 Anxiety**			
Step1: Age, gender	0.030	0.030	9.44 ***
Step 2: YPI-R3 scales	0.093	0.063	4.21 ***
**DASS-21 Stress**			
Step1: Age, gender	0.035	0.035	11.32 ***
Step 2: YPI-R3 scales	0.134	0.098	6.89 ***
**SWLS**			
Step1: Age, gender	0.009	0.009	2.82
Step 2: YPI-R3 scales	0.072	0.063	4.10 ***
**Humor Affiliative**			
Step1: Age, gender	0.001	0.001	0.21
Step 2: YPI-R3 scales	0.038	0.037	2.35 *
**Humor Aggressive**			
Step1: Age, gender	0.012	0.012	3.75 *
Step 2: YPI-R3 scales	0.030	0.018	1.12
**Humor Self-Defeating**			
Step1: Age, gender	0.039	0.039	12.57 ***
Step 2: YPI-R3 scales	0.112	0.073	4.97 ***
**Humor Self-Enhancing**			
Step1: Age, gender	0.014	0.014	4.39 *
Step 2: YPI-R3 scales	0.050	0.036	2.31 *
**ELOCS**			
Step1: Age, gender	0.025	0.025	8.05 ***
Step 2: YPI-R3 scales	0.087	0.062	4.10 ***

* *p* ≤ 0.05; ** *p* < 0.01; *** *p* < 0.001; IPIP = International Personality Item Pool; SWLS = Satisfaction with Life Scale; ELOCS = Eating Loss of Control Scale.

## Data Availability

The data underlying the results presented in the study are available from Rakesh Rai who can be reached at rakesh.kr@ctsnet.co.

## Supplementary Materials

The following supporting information can be downloaded at: https://www.mdpi.com/article/10.3390/children9050706/s1, Text S1. Investigating differences across samples; Table S1. EFA of the initial item pool of the YPI with 204 items using the Manila sample (father, *n* = 520; mother, *n* = 538); Table S2. The 23 additional items added for the EFA using Singapore and Malaysia samples; Table S3. EFA Singapore and Malaysia samples (Singapore fathers, *n* = 592; Singapore mothers, *n* = 628; Malaysia fathers, *n* = 222; Malaysia mothers, *n* = 229); Table S4. Correlation between YPI-R3 and 18 negative schemas using the fathers’ sample from the USA *(n* = 259); Table S5. Correlation between YPI-R3 and 18 negative schemas using the fathers’ sample from South Africa (*n* = 318); Table S6. Correlation between YPI-R3 and 18 negative schemas using the fathers’ sample from Nigeria (*n* = 328); Table S7. Correlation between YPI-R3 and 18 negative schemas using the fathers’ sample from India (*n* = 277); Table S8. Correlation between YPI-R3 and 18 negative schemas using the mothers’ sample from the USA (*n* = 281); Table S9. Correlation between YPI-R3 and 18 negative schemas using the mothers’ sample from South Africa (*n* = 372); Table S10. Correlation between YPI-R3 and 18 negative schemas using the mothers’ sample from Nigeria (*n* = 344); Table S11. Correlation between YPI-R3 and 18 negative schemas using the mothers’ sample from India (*n* = 289).
