# Supplementary material for: Development and Validation of the Young Parenting Inventory (YPI-R3) for Measuring Past Deviant and Normal Variations in Parenting
Source: Children (Basel). 2022 May 11;9(5):706. doi: 10.3390/children9050706 (PMC9139321; doi:10.3390/children9050706)
Supplement: Supplementary file 1 [file children-09-00706-s001.zip › children-1696874-supplementary.pdf]

Supporting Information

Development and Validation of the Young Parenting Inventory (YPI-R3) for Measuring Past Deviant Parenting Patterns

Text S1. Investigating Differences Across Samples

Explanation regarding test and calculations

A t-test is applied only to compare average values between two groups. When we compare two proportions, a z-test is the most suitable and was especially developed for this purpose. When we need to compare proportions between several groups (like race proportions in men and women) a chi-squared test is suitable.

Differences in ages between men and women using t-tests

There were significant differences in the USA sample between the age of men (M=45.04, SD=9.60), and women (M=42.90, SD=8.75), t(394=2.27, *p*=0.024.

There were significant differences in the South Africa sample between the ages of men (M=43.42 SD=6.39), and women (M=41.22, SD=6.92), t(388=3.18, *p*=0.002.

There were significant differences in the Nigeria sample between the ages of men (M=46.63, SD=7.04), and women (M=44.43, SD=7.21), t(362)=2.92, *p*=0.004.

There were no significant differences in the India sample between the ages of men (M=42.95, SD=7.33), and women (M=41.69, SD=8.05), t(304=1.43, *p*=0.154.

There were no significant differences in the Singapore sample between the ages of men (M=45.97, SD=23.27), and women (M=46.39, SD=21.69), t(626=0.31, *p*=0.756.

There was were no significant differences in the Kuala Lumpur sample between the ages of men (M=39.86, SD=17.47), and women (M=42.25, SD=17.34), t(227=1.14, *p*=0.256.

Differences in sample sizes between men and women using z-tests

USA

A z-test was conducted in the USA sample comparing the differences in the final sample size between men and women. The result was statistically significant (z=4.96, $p < 0.001$, two tailed).

A z-test was conducted in the USA sample comparing the differences in the final fathers sample size between men and women. The result was statistically significant (z=4.25, $p < 0.001$, two tailed).

A z-test was conducted in the USA sample comparing the differences in the final mothers sample size between men and women. The result was statistically significant (z=4.43, $p < 0.001$, two tailed).

South Africa

A z-test was conducted in the South African sample comparing the differences in the final sample size between men and women. The result was statistically significant (z=3.59, $p < 0.001$, two tailed).

A z-test was conducted in the South African sample comparing the differences in the final fathers sample size between men and women. The result was statistically significant (z=3.52, $p < 0.001$, two tailed).

A z-test was conducted in the South African sample comparing the differences in the final mothers sample size between men and women. The result was statistically significant (z=2.98, $p < 0.001$, two tailed).

Nigeria

A z-test was conducted in the Nigerian sample comparing the differences in the final sample size between men and women. The result was statistically significant (z=-2.80, p=0.005, two tailed).

A z-test was conducted in the Nigerian sample comparing the differences in the final fathers sample size between men and women. The result was statistically significant (z=3.05, p=0.002, two tailed).

A z-test was conducted in the Nigerian sample comparing the differences in the final mothers sample size between men and women. The result was statistically significant (z=2.77, p=0.006, two tailed).

India

A z-test was conducted in the Indian sample comparing the differences in the final sample size between men and women. The result was not statistically significant – (z=-1.82, p=0.069, two tailed).

A z-test was conducted in the Indian sample comparing the differences in the final fathers sample size between men and women. The result was not statistically significant (z=1.61, p=0.106, two tailed).

A z-test was conducted in the Indian sample comparing the differences in the final mothers sample size between men and women. The result was not statistically significant (z=1.58, p=0.114, two tailed).

Singapore

A z-test was conducted in the Singaporean sample comparing the differences in the final sample size between men and women. The result was statistically significant (z=4.32, $p < 0.001$, two tailed).

A z-test was conducted in the Singaporean sample comparing the differences in the final fathers sample size between men and women. The result was statistically significant (z=3.89, $p < 0.001$, two tailed).

A z-test was conducted in the Singaporean sample comparing the differences in the final mothers sample size between men and women. The result was statistically significant (z=4.32, $p < 0.001$, two tailed).

Kuala Lumpur

A z-test was conducted in the Kuala Lumpur sample comparing the differences in the final sample size between men and women. The result was statistically significant zZ=4.13, $p < 0.001$, two tailed).

A z-test was conducted in the Kuala Lumpur sample comparing the differences in the final fathers sample size between men and women. The result was statistically significant (z=4.13, $p < 0.001$, two tailed).

A z-test was conducted in the Kuala Lumpur sample comparing the differences in the final mothers sample size between men and women. The result was statistically significant (z=4.13, $p < 0.001$, two tailed).

Differences in races between men and women using chi-squared ($\chi 2$) tests

USA
A chi-squared test was conducted to see differences in gender regarding the various races in the USA sample. The result was not statistically significant $\chi 2$ (4, 396) = 4.48, p=0.345.

South Africa
A chi-squared test was conducted to see differences in gender regarding the various races in the South Africa sample. The result was not statistically significant $\chi 2$ (5, 390) = 1.07, p=0.899.

Nigeria
A chi-squared test was conducted to see differences in gender regarding the various races in the Nigerian sample. The result was not statistically significant $\chi 2$ (3, 364) = 3.58, p=0.311.

India
A chi-squared test was conducted to see differences in gender regarding the various races in the Nigerian sample. The result was not statistically significant $\chi 2$ (4, 306) = 4.78, p=0.312.

Singapore
A chi-squared test was conducted to see differences in gender regarding the various races in the Singapore sample. The result was not statistically significant $\chi 2$ (5, 628) = 4.78, p=0.312.

Kuala Lumpur
A chi-squared test was conducted to see differences in gender regarding the various races in the Kuala Lumpur sample. The result was not statistically significant $\chi 2$ (5, 229) = 2.43, p=0.787.

Table S1. EFA of the Initial Item Pool of the YPI with 204 Items Using Manila Sample (Father, n = 520; Mother, n = 538; Table taken from Louis et al., 2018, [19])

		Father			Mother		
RQ1 Item No.	Item Description	Loading	Selected for YPI-R3	Remarks	Loading	Selected for YPI-R3	Remarks
	Degradation and Rejection						
290	Would call me names (like "stupid" or "idiot") when I made mistakes.	.73	✓		.65	✓	
313	Saw me as lacking common sense.	.78	✓		.72	✓	
331	Put me down and made me feel ashamed of myself if I didn't do well.	.78	✓		.77	✓	
384	Saw me as having little to contribute.	.78	✓		.68	✓	
284	Treated me as if I was stupid or untalented.	.81	✓		.68	✓	
275	Made me feel like the "black sheep" of the family.	.69		Items dropped as "black sheep" may not be understood in other cultures	.58		
307	Made me feel unloved or rejected.	.65	✓		.70	✓	
386	Criticized me a lot.	.68			.75		
299	Made me feel that almost nothing I did was quite good enough.	.41			.46		
336	Was a fearful or phobic person.	.42					
363	Would ignore me or withdraw from me for long periods of time.	.43			.48		
388	Made me feel guilty if I did not put his/her needs ahead of mine.	.43					
355	I never knew how s/he was going to treat me when s/he woke the next morning.	.46			.40		
314	Often obsessed over minor decisions, because the consequences of making a mistake seemed so serious.	.47			.45		
394	Was demanding; expected to get things his/her way.	.47					
140	Was never proud of me.	.48			.45		
227	Treated me as if my opinions or desires didn't count.	.49			.46		
6	Expected me to be a failure in life.	.49			.47		
253	Would often compare my performance at school or sports unfavorably to others.	.52			.51		
407	Would withdraw from me or reject me if I did not do what s/he thought I should.	.53					
188	Often told me there was something wrong with me.	.53			.61		
281	Even if I did very well, s/he would focus on the mistakes or on things I didn't do very well.	.53			.65		
243	Down played my successes.	.54			.48		
393	Even when things were good I was always waiting for the next outburst or bad reaction from him/her.	.55			.65		
399	Did not seem to be interested in what I would do with my life.	.56			.49		
167	Often told me I was bad.	.57			.60		
380	Was unsure about my ability to reach challenging goals.	.57			.49		

Table S1 (Continued)

RQ1 Item No.	Item Description	Father Loading	Father Selected for YPI-R2	Father Remarks	Mother Loading	Mother Selected for YPI-R2	Mother Remarks
	Degradation and Rejection (Continued)						
325	I had to compete with my sibling(s) for his/her attention through outperforming them in sports or school.	.61			.63		
324	Didn't trust my ability to solve every day problems on my own that other children my age could.	.62			.65		
335	Treated me as if I was not capable of coping well on my own.	.64			.60		
383	Would make me look foolish or put me down in front of my friends or other adults.	.68			.67		
18	Believed that I was better than other people.				-.45		
120	Treated me as if there was something wrong with me.				.49		
202	Made me feel to blame when things went wrong.				.47		
293	Was critical of my friends.				.43		
303	I felt like I needed to walk on eggshells around him/her.				.44		
333	I didn't expect him/her to respect me or take my feelings into account.				.46		
	Competitiveness and Status Seeking						
98	Placed strong emphasis on success and competition.	.78	✓		.77	✓	
64	Believed that if I was smarter or more talented it made me superior to others who were less so.	.69	✓		.64	✓	
63	Put a lot of emphasis on my getting good grades and getting ahead in life.	.68	✓		.73	✓	
110	Believed that you are either a winner or a loser in life.	.63			.47		
52	Was concerned with social status and appearance.	.57	✓		.49	✓	
225	Expected me to do my best at all times.	.53	✓		.52	✓	
121	Put more emphasis on competition and winning than getting along with others.	.54			.53		
330	Drove me to excel at important tasks, couldn't settle for "good enough".	.54			.49		
236	Believed I should to what ever it takes to come out ahead.	.54			.47		
160	Was concerned with how my behavior would reflect on him/her in the eyes of others.	.51			.41		
152	Saw it a "dog eat dog" world and believed that only the toughest and best survive.	.50			.43		
172	Believed that those who come out ahead should be granted special privileges and not have to live by the same rules as others.	.46					
109	Believed that if someone had a lot of money and status that they would be happier than those who didn't.	.46					
387	Put a lot of pressure on me to excel in important areas.	.45					
198	Was a perfectionist in many areas; things had to be "just so".	.44					
244	Expected me to be the best in important areas; couldn't accept my being second best.	.43			.45		
397	Seemed to love me more or pay more attention to me when I excelled.	.42					
7	Had very high expectations for him/herself.				.47		
39	Was focused on my doing well at school and being responsible and had little interest in my having time for play and pursuing my own interests.				.43		

Table S1 (Continued)

RQ1 Item No.	Item Description	Father Loading	Father Selected for YPI-R2	Father Remarks	Mother Loading	Mother Selected for YPI-R2	Mother Remarks
	Emotional Inhibition and Deprivation						
273	Was uncomfortable expressing affection.	.74	✓		.65	✓	
302	Was private; rarely discussed his/her feelings.	.68	✓		.55	✓	
16	Had a hard time being playful.	.59	✓		.54	✓	
131	Was uncomfortable expressing his/her feelings to others; even to people s/he knew well.	.58	✓		.50	✓	
319	Did not seem comfortable playing with me.	.55	✓		.54	✓	
271	Felt uncomfortable being silly and child-like.	.50			.44		
170	Did not have a sense of humor.	.48	✓		.76	✓	
154	Was cold and distant.	.52			.42		
219	Was not available for cuddling.	.52			.49		
254	We were emotionally distant and had a hard time understanding each other.	.52					
90	I did not feel like I could go to him/her with questions about personal things; I would turn to friends or just keep it to myself.	.49					
66	Didn't seem to be interested in spending time with me.	.44					
112	Was not interested in spending unplanned time together.	.41					
85	Was too self-conscious to show positive feelings to others even when s/he wanted to.	.41					
233	Took no interest in my friends.	.41			.50		
84	Was a loner.				.51		
169	Had no interest in being part of a community.				.56		
	Undependability and Irresponsibility						
187	Was more focused on having fun and relaxing than keeping up with responsibilities.	.69	✓		.42	✓	
12	Was an undisciplined person.	.63	✓		.57	✓	
392	Spent too much money and was often in debt.	.58	✓		.44	✓	
104	Was undependable and often did not follow through on plans we made.	.53	✓		.60	✓	
221	Took money or a possession from me against my wishes to use for him/herself.	.44	✓		.52	✓	
53	Was unable to handle many daily responsibilities, so I had to do more than my share.	.48	✓		.60	✓	
155	Was an alcoholic or addicted to drugs.	.55			.56		
142	Lied to me, deceived me, or betrayed me.	.45			.51		
91	Abandoned me or left me on an emotional level when I was a child even though he/she was still physically present.	.45					
56	Provided very little discipline or structure for me.	.44					
310	Withdrew or left me alone for extended periods.	.43					
19	S/he dropped out of school and was not successful at work.	.42					
10	Never taught me the discipline necessary to succeed in school.				.50		
45	Left the house permanently when I was a child and did not keep in touch with me.				.62		
58	Would regularly break his/her promises to me.				.47		
65	I did not respect him/her.				.41		
70	Seemed to get pleasure out of hurting me.				.44		
74	Didn't really want me to succeed.				.52		
93	Didn't seem to care what happened to me.				.43		
156	Used me or took advantage of me.				.62		

Table S1 (Continued)

RQ1 Item No.	Item Description	Father Loading	Father Selected for YPI-R2	Father Remarks	Mother Loading	Mother Selected for YPI-R2	Mother Remarks
	Overprotection and Overindulgence						
283	Did a lot of things for me because s/he didn't want me to get hurt.	.66			.43		
9	Did too many things for me instead of letting me do things on my own.	.66	✓		.56	✓	
123	Overprotected me.	.62	✓		.56	✓	
95	If I didn't feel like doing a difficult or unpleasant task, I could usually get him/her to do it for me.	.61	✓		.50	✓	
4	Worried excessively that I would get hurt.	.45					
48	Worried excessively that I would get sick.	.45					
371	Spoiled me, or was overindulgent, in many respects.	.60	✓		.49	✓	
305	Treated me as if I was fragile.	.52			.46		
277	Made many decisions for me because s/he wanted to be sure things would turn out well.	.53					
366	Was over-involved in my life.	.53			.42		
49	Would do my homework for me, if I felt overwhelmed by it.	.49			.41		
311	I always let him/her make choices for me.	.46					
50	Has tried to live through me and did not allow me to be free to live my own life.	.44					
288	Treated me as if I were younger than I really was.	.43					
	Punitiveness and Abuse						
203	Would punish me when I did something wrong.	.83	✓		.65	✓	
215	Would punish me harshly when I did something wrong.	.77			.65		
148	He/she relied more on punishment than praise and rewards.	.49	✓		.45	✓	
3	Abused me physically: did things like hitting me or throwing things at me.	.49	✓		.60	✓	
46	Abused me verbally: did things like calling me names, screaming at me, swearing at me, or threatening me.	.45	✓		.54	✓	
129	His/her punishments were often out of proportion with the "crime".	.60	✓				
209	When we disagreed s/he always needed to be right.	.59	✓				
184	Everything had to be on his/her terms.	.56	✓				
168	S/he believed that if you spared the rod you spoiled the child.	.53	✓				
265	If I did what I wanted I was only asking from trouble with him/her.	.45					
263	Had to have everything under control.	.42					
	Intrusiveness and Exploitation						
156	Used me or took advantage of me.	.48	✓				
70	Seemed to get pleasure out of hurting me.	.47	✓				
256	Abused me sexually.	.44	✓				
74	Didn't really want me to succeed.	.41	✓				
	Social Exclusion						
8	Was unhappy a lot and relied on me for support and understanding.	.54	✓				
214	Discouraged me from inviting friends to our house.	.50	✓				
293	Was critical of my friends.	.49	✓				
99	Was often anxious and relied on me for reassurance and support.	.48	✓				
130	Was (seemed to be) jealous of my friends.	.47					
5	Made me feel guilty if I did not share everything with him/her.	.41					

Table S1 (Continued)

RQ1 Item No.	Item Description	Father Loading	Father Selected for YPI-R2	Father Remarks	Mother Loading	Mother Selected for YPI-R2	Mother Remarks
	Over-Control						
389	Would make me feel guilty if I did not go along with him/her.				.69	✓	
388	Made me feel guilty if I did not put his/her needs ahead of mine.				.65	✓	
394	Was demanding; expected to get things his/her way.				.51	✓	
349	Put a lot of pressure on me to meet all of my responsibilities.				.40	✓	
	Fear of Harm & Illness						
178	Worried about me or him/her being attacked.				.53	✓	
229	Worried when making decisions that something terrible would happen if s/he made the wrong choice.				.47	✓	
257	Worried about my developing a serious illness even though nothing serious was diagnosed by a physician.				.46	✓	
179	S/he often thought that something bad was likely to happen to me.				.45	✓	
248	Would worry that something terrible would happen if I made the wrong choice.				.43		
	**Total Number of items (Factors)**		**43 (8 factors)**			**39 (8 factors)**	

Total Number of items selected (Fathers)	43
Total Number of items selected (Mothers)	39
Minus: Number of overlapped items	-31
Final number of items	51
Additional Items	23
**Total no items for EFA using Singapore sample**	**74**

Table S2. The 23 Additional Items Added for the EFA Using Singapore and Kuala Lumpur Samples

Description of New Items	Construct to be Strengthened
1. When s/he held me it often felt smothering.	Intrusiveness and Exploitation
2. When s/he touched me it often felt intrusive.	Intrusiveness and Exploitation
3. Touched or held me in ways that made me feel uncomfortable or uneasy.	Intrusiveness and Exploitation
4. Left me too much on my own to make important decisions and figure things out.	Overindulgence
5. Did not help me think through the consequences of my choices.	Overindulgence
6. Left me on my own to set goals and follow through on tasks.	Overindulgence
7. Set few rules or responsibilities for me.	Overindulgence
8. Usually let me get my way.	Overindulgence
9. Put me on a pedestal.	Overindulgence
10. I could usually talk him/her into letting me do or have what I wanted.	Overindulgence
11. If I didn't feel like helping out around the house, s/he would overlook it.	Overindulgence
12. Usually put my needs ahead of everyone else's.	Overindulgence
13. Usually gave me whatever I wanted for fear that I would be angry or unhappy.	Overindulgence
14. Pressured me to follow through on tasks.	Over-Control
15. Wanted a lot of input on the goals I set.	Over-Control
16. Set a lot of rules and responsibilities for me.	Over-Control
17. Kept our family isolated from others.	Social Exclusion
18. Was a loner.	Social Exclusion
19. Had no interest in being part of a community.	Social Exclusion
20. Believed it was more important for me to gain wealth and status than be true to myself.	Competitiveness and Status Seeking
21. Never taught me the discipline necessary to succeed in school.	Undependability and Irresponsibility
22. Left the house permanently when I was a child and did not keep in touch with me.	Undependability and Irresponsibility
23. Provided very little discipline or structure for me.	Undependability and Irresponsibility

Table S3. EFA Singapore and Kuala Lumpur Samples (Singapore Father, *n* = 592; Singapore Mother, *n* = 628; Kuala Lumpur Father, *n* = 222; Kuala Lumpur Mother, *n* = 229)

RQRN Item No.	Item Description	Father		Mother		Item Selected for YPI-R3
		Singapore	Kuala Lumpur	Singapore	Kuala Lumpur	
	Over-Control					
RQRN 58	When we disagreed s/he always needed to be right.	.880		.734		✓
RQRN 38	Everything had to be on his/her terms.	.805		.790		✓
RQRN 31	Was demanding; expected to get things his/her way.	.799		.737		✓
RQRN 68	Would make me feel guilty if I did not go along with him/her.	.750		.623		✓
RQRN 69	Would call me names (like "stupid" or "idiot") when I made mistakes.	.716		.865		
RQRN 17	Made me feel guilty if I did not put his/her needs ahead of mine.	.651				✓
RQRN 35	Put a lot of pressure on me to meet all of my responsibilities.	.641				✓
	Emotional Inhibition & Deprivation					
RQRN 43	Had a hard time being playful.	.748		.888	.676	✓
RQRN 7	Was uncomfortable expressing affection.	.693			.595	✓
RQRN 42	Did not have a sense of humor.	.686		.777	.551	✓
RQRN 16	Was private; rarely discussed his/her feelings.	.613			.414	✓
RQRN 48	Was uncomfortable expressing his/her feelings to others; even to people s/he knew well.	.531			.498	✓
RQRN 27	Did not seem comfortable playing with me.	.492				✓
	Undependability and Irresponsibility					
RQRN 37	Spent too much money and was often in debt.	.828	.694	.674	.747	✓
RQRN 45	Was an undisciplined person.	.713	.735	.571	.724	✓
RQRN 33	Was undependable and often did not follow through on plans we made.	.488	.531		.533	✓
RQRN 40	Was more focused on having fun and relaxing than keeping up with responsibilities.	.479	.536			✓
RQRN 41	Took money or a possession from me against my wishes to use for him/herself.	.406		.458		✓

Table S3 (Continued)

RQRN Item No.	Item Description	Father		Mother		Item Selected for YPI-R3
		Singapore	Kuala Lumpur	Singapore	Kuala Lumpur	
	Overprotection and Overindulgence					
RQRN 29	If I didn't feel like doing a difficult or unpleasant task, I could usually get him/her to do it for me.	.681	.604	.564	.635	✓
RQRN 51	Did too many things for me instead of letting me do things on my own.	.613	.526	.656	.667	✓
RQRN 59	Spoiled me, or was overindulgent, in many respects.	.600	.793	.752	.581	✓
RQRN 30	I could usually talk him/her into letting me do or have what I wanted.	.559				✓
RQRN 63	Overprotected me.	.503	.486	.510	.448	✓
RQRN 65	Usually gave me whatever I wanted for fear that I would be angry or unhappy.	.503	.627	.573	.461	✓
	Neglect and Insufficient Guidance					
RQRN 44	Left me on my own to set goals and follow through on tasks.	.832	.503	.575		✓
RQRN 56	Left me too much on my own to make important decisions and figure things out.	.741	.530	.824		✓
RQRN 25	Usually let me get my way.	.424	.698			✓
RQRN24	Never taught me the discipline necessary to succeed in school.		.519	.480		✓
RQRN13	Provided very little discipline or structure for me.		.512	.437		✓
	Competitiveness and Status Seeking					
RQRN 3	Placed strong emphasis on success and competition.	.761	.783	.671	.854	✓
RQRN 9	Put a lot of emphasis on my getting good grades and getting ahead in life.	.616	.861	.627	.936	✓
RQRN 21	Believed that if I was smarter or more talented it made me superior to others who were less so.	.481	.640	.459	.777	✓
RQRN36	Was concerned with social status and appearance.		.695	.585	.693	✓

Table S3 (Continued)

RQRN Item No.	Item Description	Father Singapore	Father Kuala Lumpur	Mother Singapore	Mother Kuala Lumpur	Item Selected for YPI-R3
	Fear of Harm and Illness					
RQRN 62	Was often anxious and relied on me for reassurance and support.	.710	.551		.643	✓
RQRN 52	Was unhappy a lot and relied on me for support and understanding.	.525			.455	✓
RQRN 10	Worried about me or him/her being attacked.	.519	.639	.727	.480	✓
RQRN 50	Worried about my developing a serious illness even though nothing serious was diagnosed by a physician.	.492	.665	.741	.558	✓
RQRN 22	Worried when making decisions that something terrible would happen if s/he made the wrong choice.	.472	.463	.449		✓
RQRN 61	S/he often thought that something bad was likely to happen to me.		.484	.475		✓
	Intrusiveness and Exploitation					
RQRN 67	Touched or held me in ways that made me feel uncomfortable or uneasy.	.711		.648	.681	✓
RQRN 14	When s/he touched me it often felt intrusive.	.631		.776	.476	✓
RQRN 11	Abused me sexually.	.419			.592	✓
RQRN 15	Used me or took advantage of me.	.408			.420	✓
RQRN 20	Seemed to get pleasure out of hurting me.				.613	✓
	Degradation and Rejection					
RQRN 74	Saw me as lacking common sense.	.622	.601			✓
RQRN 12	Saw me as having little to contribute.	.585				✓
RQRN 18	Treated me as if I was stupid or untalented.	.578	.694			✓
RQRN 26	Made me feel unloved or rejected.					✓
RQRN 1	Put me down and made me feel ashamed of myself if I didn't do well.		.655			✓
RQRN 69	Would call me names (like "stupid" or "idiot") when I made mistakes.		.816			✓
	Social Exclusion					
RQRN 55	Was a loner.	.783	.747	.687		✓
RQRN 71	Had no interest in being part of a community.	.693	.472	.614	.458	✓
RQRN 4	Kept our family isolated from others.	.609	.481	.466	.626	✓
	Punitiveness and Abuse					
RQRN 19	Would punish me when I did something wrong.	.496	.703	.510	.702	✓
RQRN 5	S/he believed that if you spared the rod you spoiled the child.	.489		.566	.779	✓
RQRN 28	He/she relied more on punishment than praise and rewards.	.419			.489	✓
RQRN 47	Abused me verbally: did things like calling me names, screaming at me, swearing at me, or threatening me.		1.129		.714	✓
RQRN 34	Abused me physically: did things like hitting me or throwing things at me.		.694		.659	✓

Note. Total number of items = 57, Total number of subscales = 11; Final number of items selected = 41, Final number of subscales = 10 — 57 items

Table S4. Correlation between YPI-R3 and 18 Negative Schemas Using Fathers Sample from USA (*n*=259)

	OC	EID	UI	OO	NIG	CSS	IE	DR	SE	PA
Abandonment	.159*	.021	.223**	.225**	.185**	.032	.145*	.139*	.073	.090
Approvalseeking	.129*	.143*	.121	.252**	.149*	.162**	.072	.124*	.130*	.068
Defectiveness	.119	.133*	.081	.159*	.131*	.022	.129*	.209**	.153*	.180**
Dependence	.064	.091	.148*	.234**	.145*	-.057	.105	.116	.064	.062
Emotionaldeprivation	.169**	.127*	.099	.114	.135*	.081	.177**	.160*	.199**	.141*
Emotionalinhibition	.007	.161**	.065	.159*	.189**	.059	.071	.020	.190**	.005
Enmeshment	.062	-.042	.049	.199**	.003	-.058	.090	.045	.048	-.013
Entitlement	.006	.008	.129*	.263**	.082	.066	.028	-.040	.025	-.040
Failure	.099	.103	.136*	.146*	.137*	-.090	.079	.106	.087	.057
Insufficient Self-Control	.073	.143*	.179**	.258**	.262**	-.023	.128*	.098	.170**	.088
Mistrust	.117	.096	.181**	.193**	.175**	.071	.092	.120	.125*	.099
Pessimism	.174**	.118	.227**	.143*	.213**	.115	.162**	.141*	.186**	.138*
Puniiveness	.127*	.110	.046	.111	.092	.120	.064	.081	.103	.080
Selfsacrifice	.075	.063	.128*	.133*	.084	.109	-.012	.007	.076	-.011
SocialIsolation	.128*	.152*	.108	.174**	.143*	.012	.018	.128*	.198**	.094
Subjugation	.077	.138*	.167**	.131*	.180**	.031	.033	.065	.218**	.037
Unrelenting Standards	.125*	.118	.052	.100	.025	.191**	-.050	.007	.121	.031
Vulnerability	.158*	.090	.165**	.164**	.195**	.096	.151*	.129*	.150*	.123*

OC = Over-Control; EID = Emotional Inhibition and Deprivation; UI = Undependability and Irresponsibility; OO = Overprotection and Overindulgence; NIG – Neglect and Insufficient Guidance; CSS = Competitiveness and Status Seeking; IE = Intrusiveness and Exploitation; DR = Degradation and Rejection; SE = Social Exclusion; PA = Punitiveness and Abuse

Table S5. Correlation between YPI-R3 and 18 Negative Schemas Using Fathers Sample from South Africa (*n*=318)

	OC	EID	UI	OO	NIG	CSS	IE	DR	SE	PA
Abandonment	.121*	.083	.180**	.258**	.192**	.162**	.150**	.174**	.094	.117*
Approvalseeking	.171**	.184**	.168**	.223**	.184**	.175**	.163**	.154**	.129*	.115*
Defectiveness	.267**	.291**	.279**	.247**	.305**	.110*	.308**	.299**	.267**	.264**
Dependence	.194**	.220**	.224**	.262**	.254**	.178**	.147**	.275**	.149**	.157**
Emotionaldeprivation	.172**	.226**	.216**	.152**	.277**	.111*	.196**	.213**	.224**	.209**
Emotionalinhibition	.203**	.284**	.129*	.204**	.172**	.144*	.097	.167**	.246**	.146**
Enmeshment	.143*	.127*	.226**	.244**	.194**	.169**	.200**	.256**	.077	.141*
Entitlement	.163**	.150**	.129*	.301**	.147**	.215**	.117*	.167**	.110	.158**
Failure	.170**	.209**	.232**	.219**	.290**	.100	.168**	.275**	.223**	.181**
Insufficient Self-Control	.189**	.223**	.180**	.299**	.280**	.167**	.085	.180**	.186**	.161**
Mistrust	.246**	.181**	.222**	.271**	.258**	.173**	.224**	.237**	.250**	.212**
Pessimism	.208**	.223**	.227**	.237**	.251**	.188**	.189**	.213**	.220**	.186**
Puniiveness	.291**	.240**	.207**	.205**	.294**	.171**	.221**	.263**	.255**	.228**
Selfsacrifice	.170**	.159**	.216**	.234**	.190**	.154**	.078	.168**	.171**	.108
SocialIsolation	.246**	.267**	.163**	.192**	.276**	.095	.167**	.212**	.287**	.198**
Subjugation	.246**	.218**	.246**	.247**	.287**	.150**	.192**	.233**	.209**	.145**
Unrelenting Standards	.292**	.150**	.133*	.222**	.155**	.309**	.160**	.206**	.150**	.184**
Vulnerability	.287**	.220**	.309**	.190**	.222**	.199**	.255**	.242**	.188**	.226**

OC = Over-Control; EID = Emotional Inhibition and Deprivation; UI = Undependability and Irresponsibility; OO = Overprotection and Overindulgence; NIG – Neglect and Insufficient Guidance; CSS = Competitiveness and Status Seeking; IE = Intrusiveness and Exploitation; DR = Degradation and Rejection; SE = Social Exclusion; PA = Punitiveness and Abuse

Table S6. Correlation between YPI-R3 and 18 Negative Schemas Using Fathers Sample from Nigeria (*n*=328)

	OC	EID	UI	OO	NIG	CSS	IE	DR	SE	PA
Abandonment	.195**	.155**	.258**	.314**	.253**	.255**	.306**	.184**	.221**	.115*
Approvalseeking	.192**	.183**	.263**	.238**	.238**	.267**	.265**	.185**	.159**	.102
Defectiveness	.191**	.226**	.268**	.352**	.269**	.226**	.310**	.227**	.269**	.112*
Dependence	.212**	.202**	.266**	.359**	.286**	.194**	.277**	.179**	.303**	.130*
Emotionaldeprivation	.231**	.295**	.298**	.215**	.364**	.163**	.310**	.251**	.272**	.218**
Emotionalinhibition	.253**	.311**	.320**	.262**	.332**	.184**	.325**	.312**	.326**	.202**
Enmeshment	.208**	.171**	.269**	.347**	.217**	.241**	.321**	.210**	.242**	.115*
Entitlement	.256**	.260**	.258**	.282**	.276**	.277**	.264**	.191**	.263**	.175**
Failure	.197**	.143**	.215**	.339**	.213**	.209**	.280**	.179**	.216**	.111*
Insufficient Self-Control	.176**	.237**	.242**	.288**	.307**	.228**	.254**	.201**	.262**	.147**
Mistrust	.236**	.209**	.274**	.252**	.310**	.267**	.245**	.215**	.259**	.161**
Pessimism	.219**	.200**	.271**	.289**	.261**	.295**	.259**	.206**	.252**	.132*
Puniiveness	.186**	.127*	.231**	.285**	.202**	.244**	.244**	.188**	.185**	.105
Selfsacrifice	.108	.111*	.171**	.150**	.212**	.159**	.139*	.103	.205**	.058
SocialIsolation	.229**	.266**	.265**	.259**	.343**	.185**	.290**	.221**	.334**	.166**
Subjugation	.273**	.227**	.284**	.312**	.276**	.309**	.334**	.263**	.288**	.183**
Unrelenting Standards	.226**	.190**	.194**	.205**	.231**	.285**	.168**	.144**	.202**	.140*
Vulnerability	.222**	.251**	.262**	.258**	.258**	.215**	.256**	.219**	.258**	.189**

OC = Over-Control; EID = Emotional Inhibition and Deprivation; UI = Undependability and Irresponsibility; OO = Overprotection and Overindulgence; NIG – Neglect and Insufficient Guidance; CSS = Competitiveness and Status Seeking; IE = Intrusiveness and Exploitation; DR = Degradation and Rejection; SE = Social Exclusion; PA = Punitiveness and Abuse

Table S7. Correlation between YPI-R3 and 18 Negative Schemas Using Fathers Sample from India (*n*=277)

	OC	EID	UI	OO	NIG	CSS	IE	DR	SE	PA
Abandonment	.320**	.161**	.403**	.372**	.236**	.241**	.444**	.246**	.233**	.188**
Approvalseeking	.256**	.152*	.259**	.308**	.245**	.233**	.213**	.173**	.135*	.100
Defectiveness	.244**	.180**	.344**	.377**	.189**	.218**	.372**	.261**	.233**	.140*
Dependence	.239**	.170**	.402**	.388**	.243**	.188**	.408**	.260**	.196**	.189**
Emotionaldeprivation	.211**	.213**	.249**	.173**	.164**	.140*	.327**	.202**	.195**	.126*
Emotionalinhibition	.221**	.171**	.250**	.274**	.116	.165**	.238**	.128*	.272**	.135*
Enmeshment	.269**	.070	.451**	.511**	.156**	.205**	.442**	.233**	.251**	.182**
Entitlement	.241**	.123*	.263**	.337**	.078	.209**	.270**	.191**	.203**	.095
Failure	.258**	.223**	.318**	.348**	.244**	.197**	.339**	.307**	.227**	.147*
Insufficient Self-Control	.230**	.149*	.259**	.328**	.197**	.155**	.256**	.177**	.107	.089
Mistrust	.268**	.210**	.408**	.346**	.226**	.205**	.320**	.205**	.348**	.207**
Pessimism	.252**	.118*	.313**	.251**	.160**	.167**	.259**	.180**	.165**	.144*
Puniiveness	.202**	.085	.258**	.285**	.177**	.219**	.249**	.184**	.142*	.121*
Selfsacrifice	.154*	.064	.217**	.220**	.100	.142*	.159**	.056	.213**	.083
SocialIsolation	.255**	.250**	.360**	.287**	.184**	.175**	.337**	.233**	.290**	.173**
Subjugation	.243**	.116	.324**	.349**	.222**	.151*	.306**	.246**	.192**	.123*
Unrelenting Standards	.250**	.184**	.270**	.253**	.164**	.250**	.258**	.175**	.295**	.148*
Vulnerability	.212**	.139*	.298**	.346**	.116	.149*	.304**	.172**	.198**	.137*

OC = Over-Control; EID = Emotional Inhibition and Deprivation; UI = Undependability and Irresponsibility; OO = Overprotection and Overindulgence; NIG – Neglect and Insufficient Guidance; CSS = Competitiveness and Status Seeking; IE = Intrusiveness and Exploitation; DR = Degradation and Rejection; SE = Social Exclusion; PA = Punitiveness and Abuse

Table S8. Correlation between YPI-R3 and 18 Negative Schemas Using Mothers Sample from USA (*n*=281)

	OC	EID	UI	OO	IGN	CSS	IE	DR	SE	PA
Abandonment	.272**	.126*	.234**	.238**	.195**	.048	.233**	.253**	.132*	.196**
Approvalseeking	.176**	.151*	.175**	.254**	.164**	.198**	.034	.139*	.107	.050
Defectiveness	.222**	.187**	.229**	.155**	.233**	.090	.233**	.236**	.231**	.193**
Dependence	.158**	.175**	.147*	.234**	.202**	.026	.191**	.231**	.153*	.135*
Emotionaldeprivation	.167**	.186**	.206**	.112	.233**	.192**	.219**	.250**	.206**	.182**
Emotionalinhibition	.162**	.270**	.141*	.208**	.245**	.148*	.124*	.105	.165**	.067
Enmeshment	.168**	.125*	.158**	.259**	.141*	.045	.257**	.201**	.171**	.095
Entitlement	.102	.072	.084	.235**	.120*	.161**	.077	.104	.076	.004
Failure	.186**	.146*	.163**	.142*	.226**	-.019	.201**	.236**	.175**	.128*
Insufficient Self-Control	.304**	.224**	.292**	.312**	.320**	.131*	.266**	.353**	.261**	.214**
Mistrust	.300**	.201**	.168**	.165**	.194**	.199**	.177**	.253**	.229**	.221**
Pessimism	.295**	.177**	.209**	.176**	.231**	.166**	.219**	.269**	.236**	.185**
Puniiveness	.275**	.174**	.129*	.130*	.179**	.195**	.178**	.224**	.158**	.149*
Selfsacrifice	.287**	.151*	.123*	.193**	.111	.120*	.059	.115	.105	.109
SocialIsolation	.282**	.196**	.182**	.255**	.208**	.179**	.134*	.198**	.218**	.130*
Subjugation	.264**	.178**	.125*	.278**	.160**	.134*	.134*	.166**	.186**	.123*
Unrelenting Standards	.208**	.146*	.039	.193**	.055	.232**	.014	.088	.118*	-.001
Vulnerability	.256**	.168**	.221**	.208**	.232**	.152*	.291**	.276**	.231**	.183**

**. Correlation is significant at the 0.01 level (2-tailed).

*. Correlation is significant at the 0.05 level (2-tailed).

OC = Over-Control; EID = Emotional Inhibition and Deprivation; UI = Undependability and Irresponsibility; OO = Overprotection and Overindulgence; NIG – Neglect and Insufficient Guidance; CSS = Competitiveness and Status Seeking; IE = Intrusiveness and Exploitation; DR = Degradation and Rejection; SE = Social Exclusion; PA = Punitiveness and Abuse

Table S9. Correlation between YPI-R3 and 18 Negative Schemas Using Mothers Sample from South Africa (*n*= 372)

	OC	EID	UI	OO	IGN	CSS	IE	DR	SE	PA
Abandonment	.247**	.183**	.266**	.202**	.274**	.203**	.320**	.292**	.273**	.227**
Approvalseeking	.189**	.142**	.197**	.271**	.191**	.205**	.144**	.189**	.196**	.123*
Defectiveness	.285**	.258**	.335**	.238**	.360**	.188**	.370**	.359**	.338**	.272**
Dependence	.220**	.172**	.324**	.282**	.325**	.188**	.302**	.265**	.171**	.205**
Emotionaldeprivation	.308**	.283**	.297**	.175**	.352**	.192**	.386**	.308**	.324**	.299**
Emotionalinhibition	.265**	.279**	.194**	.235**	.245**	.239**	.206**	.203**	.270**	.217**
Enmeshment	.264**	.101	.300**	.356**	.280**	.195**	.293**	.252**	.164**	.221**
Entitlement	.211**	.175**	.224**	.300**	.235**	.231**	.155**	.162**	.246**	.144**
Failure	.231**	.150**	.309**	.244**	.357**	.145**	.264**	.310**	.220**	.234**
Insufficient Self-Control	.191**	.146**	.235**	.339**	.293**	.178**	.144**	.165**	.204**	.164**
Mistrust	.312**	.157**	.298**	.275**	.319**	.207**	.285**	.294**	.321**	.246**
Pessimism	.218**	.125*	.252**	.297**	.263**	.184**	.209**	.204**	.165**	.129*
Puniiveness	.303**	.198**	.298**	.228**	.312**	.240**	.278**	.297**	.265**	.209**
Selfsacrifice	.219**	.146**	.234**	.251**	.191**	.217**	.171**	.177**	.197**	.134**
SocialIsolation	.268**	.237**	.259**	.222**	.314**	.181**	.264**	.258**	.339**	.201**
Subjugation	.241**	.169**	.281**	.356**	.292**	.213**	.229**	.206**	.258**	.148**
Unrelenting Standards	.335**	.222**	.215**	.237**	.229**	.337**	.166**	.219**	.221**	.209**
Vulnerability	.246**	.157**	.282**	.203**	.264**	.173**	.266**	.256**	.142**	.181**

**. Correlation is significant at the 0.01 level (2-tailed).

*. Correlation is significant at the 0.05 level (2-tailed).

OC = Over-Control; EID = Emotional Inhibition and Deprivation; UI = Undependability and Irresponsibility; OO = Overprotection and Overindulgence; NIG – Neglect and Insufficient Guidance; CSS = Competitiveness and Status Seeking; IE = Intrusiveness and Exploitation; DR = Degradation and Rejection; SE = Social Exclusion; PA = Punitiveness and Abuse

Table S10. Correlation between YPI-R3 and 18 Negative Schemas using Mothers Sample from Nigeria (*n*= 344)

	OC	EID	UI	OO	IGN	CSS	IE	DR	SE	PA
Abandonment	.205**	.169**	.259**	.364**	.241**	.332**	.299**	.192**	.297**	.135*
Approvalseeking	.208**	.186**	.190**	.317**	.182**	.358**	.252**	.185**	.278**	.149**
Defectiveness	.237**	.226**	.305**	.317**	.213**	.239**	.324**	.213**	.356**	.192**
Dependence	.157**	.173**	.291**	.339**	.234**	.203**	.308**	.196**	.334**	.134*
Emotionaldeprivation	.186**	.166**	.296**	.260**	.229**	.215**	.262**	.164**	.247**	.124*
Emotionalinhibition	.246**	.346**	.265**	.292**	.291**	.257**	.291**	.238**	.372**	.179**
Enmeshment	.238**	.211**	.293**	.279**	.219**	.277**	.359**	.227**	.355**	.219**
Entitlement	.204**	.173**	.217**	.327**	.165**	.317**	.260**	.160**	.380**	.169**
Failure	.214**	.205**	.245**	.363**	.257**	.259**	.303**	.198**	.357**	.162**
Insufficient Self-Control	.216**	.231**	.286**	.317**	.275**	.264**	.300**	.233**	.329**	.171**
Mistrust	.205**	.176**	.225**	.301**	.226**	.258**	.258**	.187**	.269**	.169**
Pessimism	.244**	.254**	.234**	.315**	.200**	.332**	.264**	.204**	.325**	.162**
Puniiveness	.242**	.162**	.236**	.279**	.207**	.354**	.293**	.170**	.268**	.159**
Selfsacrifice	.164**	.120*	.141**	.220**	.072	.201**	.175**	.117*	.173**	.166**
SocialIsolation	.247**	.268**	.324**	.315**	.228**	.212**	.279**	.198**	.364**	.151**
Subjugation	.258**	.225**	.293**	.314**	.207**	.302**	.297**	.196**	.327**	.160**
Unrelenting Standards	.214**	.155**	.132*	.265**	.143**	.311**	.151**	.109*	.267**	.159**
Vulnerability	.170**	.238**	.255**	.327**	.241**	.204**	.243**	.192**	.337**	.140**

**. Correlation is significant at the 0.01 level (2-tailed).

*. Correlation is significant at the 0.05 level (2-tailed).

OC = Over-Control; EID = Emotional Inhibition and Deprivation; UI = Undependability and Irresponsibility; OO = Overprotection and Overindulgence; NIG – Neglect and Insufficient Guidance; CSS = Competitiveness and Status Seeking; IE = Intrusiveness and Exploitation; DR = Degradation and Rejection; SE = Social Exclusion; PA = Punitiveness and Abuse

Table S11. Correlation between YPI-R3 and 18 Negative Schemas using Mothers Sample from India (*n*= 289)

	OC	EID	UI	OO	IGN	CSS	IE	DR	SE	PA
Abandonment	.307**	.232**	.413**	.372**	.271**	.285**	.408**	.323**	.295**	.255**
Approvalseeking	.221**	.189**	.295**	.288**	.222**	.264**	.210**	.196**	.174**	.161**
Defectiveness	.293**	.337**	.411**	.324**	.289**	.265**	.420**	.423**	.300**	.275**
Dependence	.268**	.250**	.414**	.346**	.319**	.235**	.422**	.387**	.269**	.238**
Emotionaldeprivation	.239**	.315**	.321**	.224**	.257**	.179**	.318**	.319**	.315**	.248**
Emotionalinhibition	.169**	.200**	.301**	.283**	.228**	.166**	.266**	.148*	.254**	.189**
Enmeshment	.274**	.221**	.424**	.419**	.245**	.161**	.443**	.283**	.373**	.231**
Entitlement	.279**	.293**	.357**	.331**	.173**	.230**	.246**	.194**	.226**	.217**
Failure	.292**	.297**	.344**	.292**	.260**	.170**	.371**	.357**	.299**	.247**
Insufficient Self-Control	.253**	.261**	.384**	.345**	.294**	.249**	.261**	.247**	.234**	.198**
Mistrust	.349**	.276**	.412**	.334**	.257**	.306**	.355**	.289**	.307**	.291**
Pessimism	.276**	.208**	.374**	.248**	.249**	.231**	.258**	.254**	.258**	.202**
Puniiveness	.242**	.111	.296**	.273**	.124*	.247**	.243**	.286**	.203**	.193**
Selfsacrifice	.262**	.163**	.229**	.200**	.170**	.146*	.146*	.171**	.216**	.193**
SocialIsolation	.271**	.305**	.338**	.301**	.244**	.205**	.352**	.333**	.309**	.256**
Subjugation	.299**	.208**	.375**	.383**	.266**	.218**	.304**	.305**	.288**	.245**
Unrelenting Standards	.299**	.301**	.258**	.302**	.209**	.278**	.249**	.243**	.222**	.236**
Vulnerability	.312**	.298**	.408**	.289**	.227**	.230**	.377**	.328**	.277**	.271**

**. Correlation is significant at the 0.01 level (2-tailed).

*. Correlation is significant at the 0.05 level (2-tailed).

OC = Over-Control; EID = Emotional Inhibition and Deprivation; UI = Undependability and Irresponsibility; OO = Overprotection and Overindulgence; NIG – Neglect and Insufficient Guidance; CSS = Competitiveness and Status Seeking; IE = Intrusiveness and Exploitation; DR = Degradation and Rejection; SE = Social Exclusion; PA = Punitiveness and Abuse
